# Thresholds of biodiversity and ecosystem function in a forest ecosystem undergoing dieback

**DOI:** 10.1038/s41598-017-06082-6

**Published:** 2017-07-28

**Authors:** P. M. Evans, A. C. Newton, E. Cantarello, P. Martin, N. Sanderson, D. L. Jones, N. Barsoum, J. E. Cottrell, S. W. A’Hara, L. Fuller

**Affiliations:** 1 Centre for Conservation Ecology and Environmental Sciences, Faculty of Science and Technology, Bournemouth University, Poole, BH12 5BB UK; 2 Botanical Survey and Assessment, 3 Green Close, Woodlands, Southampton, Hampshire SO40 7HU UK; 3 School of Environment, Natural Resources and Geography, Bangor University, Gwynedd, LL57 2UW UK; 4 Forest Research, Alice Holt Lodge, Farnham, Surrey, GU10 4LH UK; 5 Biological and Environmental Sciences, University of Stirling, Stirling, FK9 4LA UK

## Abstract

Ecological thresholds, which represent points of rapid change in ecological properties, are of major scientific and societal concern. However, very little research has focused on empirically testing the occurrence of thresholds in temperate terrestrial ecosystems. To address this knowledge gap, we tested whether a number of biodiversity, ecosystem functions and ecosystem condition metrics exhibited thresholds in response to a gradient of forest dieback, measured as changes in basal area of living trees relative to areas that lacked recent dieback. The gradient of dieback was sampled using 12 replicate study areas in a temperate forest ecosystem. Our results provide novel evidence of several thresholds in biodiversity (namely species richness of ectomycorrhizal fungi, epiphytic lichen and ground flora); for ecological condition (e.g. sward height, palatable seedling abundance) and a single threshold for ecosystem function (i.e. soil respiration rate). Mechanisms for these thresholds are explored. As climate-induced forest dieback is increasing worldwide, both in scale and speed, these results imply that threshold responses may become increasingly widespread.

## Introduction

The living world is currently experiencing an unprecedented period of environmental change^1–4^. In recent decades, human-derived actions such as carbon emission, introduction of species and large-scale land transformations (e.g. urban and agricultural expansion) have become pervasive throughout the biosphere. Impacts of human activity have become so widespread and intrusive that a new geological epoch, the Anthropocene, has been proposed^5^. Human actions have influenced the functioning of the Earth system to such an extent that the consequences could be detrimental or even catastrophic for human society^1–4^. This is reflected in development of the planetary boundaries concept, which suggests that if specific thresholds of environmental change are transgressed, there may be increased risks to human wellbeing or to resilience of the whole Earth system^2, 3^.

The concept of planetary boundaries, together with allied concepts such as resilience^2, 3^, depends on the existence of ecological thresholds. Such thresholds are defined as points or zones where relatively rapid change occurs from one ecological condition to another^6^, and are characterised by a non-linear response of an ecosystem property to a controlling variable that increases linearly^7^. If thresholds occur in nature, a slight increase in disturbance intensity or frequency could cause a disproportionate change in an ecosystem property. Such changes could include the loss of biodiversity crucial for ecosystem function^8^ and the loss of regulatory ecosystem services on which humans depend^9^. Moreover, a threshold in one ecosystem property could sequentially disrupt the self-organising networks that govern local dynamics of other systems^10^, and could potentially cause unpredictable responses at the scale of whole Earth system dynamics^3, 6, 11^. There is a need to avoid crossing such thresholds to enable ecological systems, and their associated socio-economic systems, to be maintained in the future^12^.

Ecological thresholds are thought to be attributable to shifts in the relative strength of balancing (i.e. negative) and reinforcing (i.e. positive) feedback loops that influence the dynamics of an ecosystem^13^. For example, in many terrestrial ecosystems, low water availability acts to regulate the growth of plants. Conversely, if water availability increases by a sufficient amount, the biomass and complexity of vegetation can increase, which can further increase water availability by modifying the water cycle^14, 15^.

Despite the perceived global importance of ecological thresholds, supporting evidence is largely theoretical^7, 16^, and the issue is the focus of major scientific debate^17, 18^. Supporting empirical evidence from field situations is severely limited^6, 19^, and is primarily available for aquatic systems^20–22^. Field evidence for ecological thresholds resulting from environmental change is particularly lacking in temperate woodland ecosystems that are not governed by fire^6, 23^. This research therefore aimed to test the hypothesis that threshold responses exist in measures of (1) biodiversity, (2) ecosystem function and (3) ecosystem condition within a terrestrial ecosystem, specifically temperate forest. To test this hypothesis, we examined a beech-dominated forest that is currently undergoing large-scale dieback in response to environmental change, as revealed through analysis of long-term monitoring data^24^.

## Methods

### Study area

**Figure 1:**
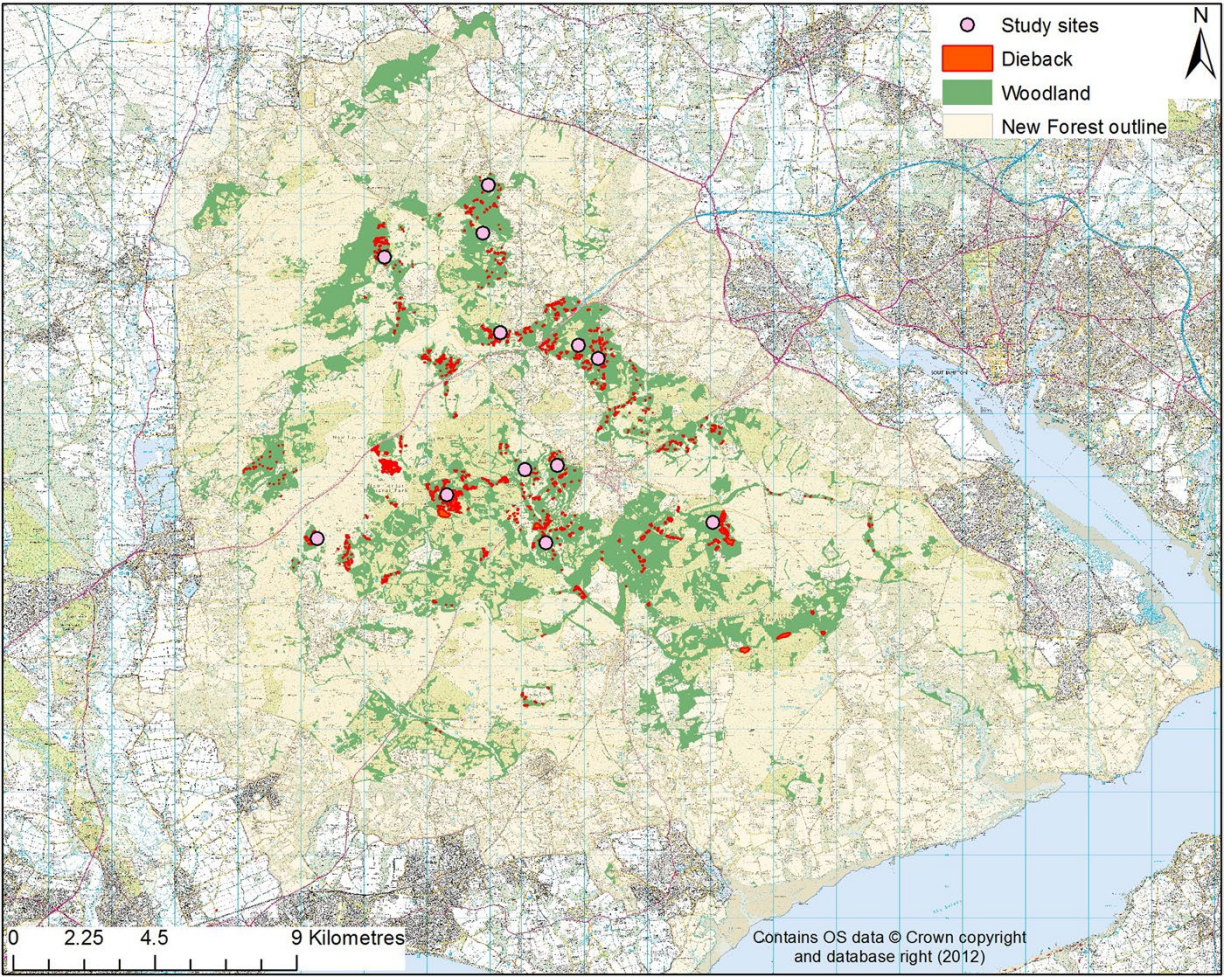
Distribution of broadleaved woodland (green), occurrence of dieback (red) and location of each of the 12 study areas (pink dots) in the New Forest, in southern England. Map was made using ArcMap 10.1 (http://desktop.arcgis.com/en/arcmap/).

We carried out this study in the New Forest National Park (NP), which covers an area of 57,100 ha situated in southern England (longitude: 1°17′59″ to 1°48′8″ W, Latitude: 50°42′19″ to 51°0′17″ N) (Fig. 1). The Forest consists of a mosaic of heathland, mire, grassland and coniferous and broadleaf woodland (8,472 ha) ecosystems. These woodlands are dominated by beech (*Fagus sylvatica*), often occurring with oak (*Quercus robur*) and birch (*Betula pendula*), and typically with holly (*Ilex aquifolium*) in the understorey^25^. The local climate is oceanic and temperate, with a mean annual maximum temperature of 14.8 °C and annual rainfall of 835.2 mm, based on data available between 1981 and 2010^26^. The Park contains the largest area of semi-natural vegetation in lowland Britain^27, 28^, and is of exceptional importance for biodiversity conservation^29^. The New Forest is also characterised by high densities of large herbivores, including livestock and deer, reflecting its history as a Royal hunting reserve^27^.

### Experimental design

A geographic information system (GIS) (ArcGIS 10.1) was utilised to identify suitable study sites of forest dieback within the New Forest. Spatial information included 25 cm resolution aerial photographs, captured in 2007 by GeoPerspectives, and areas of known historic woodland dieback, recorded in 1999^30^. The resulting areas of dieback were overlaid on top of several layers, including soil data (NATMAP; National Soil Map), obtained from National Soil Resources Institute (NSRI), Silsoe, Bedfordshire, UK; regeneration plots; and a tree composition map, derived from data collected in 1982^31^. Twelve sites where recent dieback of mature native broadleaf woodland has been observed^32^ were selected for study. Within each site, five 20 × 20 m survey plots were established to provide a gradient of woodland dieback, using basal area (BA) as a measure of forest structure, calculated following Cantarello and Newton^33^. In each case, beech was the dominant canopy tree species. Plots were situated to provide values of 100%, 75%, 50%, 25% and 0% BA (see Supplementary Information Fig. S1 and Table S1), with 100% representing an intact forest stand and 0% indicating complete death of all canopy trees, identified by the presence of standing deadwood. Secondary criteria required canopy openness due to canopy death to increase positively with dieback stage, and that plots other than the intact stage plots had standing or lying deadwood present. The five stages were: (1) intact (no dieback); (2) slight dieback; (3) moderate dieback; (4) major dieback; and (5) total dieback. The mean of the 12 intact plots was used as a baseline value. In this way, in each of the 12 study sites, one plot was sampled in each of the five basal area classes. This design represents a form of space-for-time substitution, for which spatial variation in BA was assumed to represent temporal dieback of the forest stands.

### Plot set-up

Each plot was 20 × 20 m (400 m^2^; 0.04 ha). A nested sub-plot of 10 × 10 m (100 m^2^) was set up in the centre of each plot, laid out in the same orientation as the full plot. The centre and the corners of the sub-plot were marked with wooden stakes for easy identification on return visits. The mid-points of each plot were recorded using a handheld GPS (GPSMAP 60CSx; Garmin, USA) (see Supplementary Information SM1).

### Field measurements

Within each survey plot we identified tree species and diameter at breast height (dbh, 1.3 m) were recorded. We undertook detailed surveys of each plot to identify species of epiphytic lichens, ground flora, tree saplings and seedlings and ectomycorrhizal fungi (ECM) based on the identification of sporocarps. In five sites ground-dwelling arthropods were trapped in pitfall traps and identified using DNA barcoding methods (see Supplementary Information SM2).

As soil condition and structure are important to the productivity of the whole woodland ecosystem, we sampled soils within each plot then analysed bulk density, nitrate, ammonium, potentially mineralisable nitrogen, C, K, P, S, Ca, Mg, Na, Al, Mn, pH, electrical conductivity, organic matter, soil moisture, soil temperature and particle size distribution using standard analytical procedures. We recorded *in-situ* nitrogen mineralisation and nitrification using a resin capsule (Unibest, Walla Walla, WA, USA), following DeLuca *et al*.^34^ (see Supplementary Information SM1).

We made measurements of tree crown condition^35^, canopy openness^36^ and deadwood volume following Newton^37^. As a metric of herbivory, dung counts^38^, plant browsing^39, 40^ scrub layer presence and condition^41^ and sward height^42^ were recorded. Aboveground biomass and carbon storage were calculated following Jenkins *et al*.^43^. Soil respiration rate was measured with a portable EGM-4 Environmental Gas Monitor CO_2_ infrared gas analyser (IRGA) equipped with a closed system soil respiration chamber (PP Systems, Amesbury, MA, USA) (see Supplementary Information SM1). For all variables measured, see Supplementary Information Table S2.

### Data analysis

All measured variables were analysed in relation to gradients in BA, treating the twelve sites as independent replicates. As BA was scaled linearly along the gradients (see Supplementary Information Fig. S1 and Table S1), any departure from linearity provided potential evidence of a threshold response. Generalised linear mixed models (GLMMs) were used to analyse these responses. This was achieved by fitting the most parsimonious models (determined using AICc) of the relationships between percentage BA and the response variables, using other measured predictors as fixed effects and study area as a random effect. All analyses were performed in R 3.1.2. (R Development Core Team, 2011, http://www.R-project.org) using the lme4^44^ and ggplot2^45^ packages for mixed models. In this study, the *r* ^2^ measure used was the marginal *r* ^2^, which describes the proportion of variance explained by the fixed effect alone^46^. A response variable was considered to show a threshold if it met three criteria relating to the most parsimonious model: (1) the model included a quadratic term; (2) its ΔAICc was ≥3 compared to the next closest model; and (3) its marginal *r* ^2^ value was >0.15. These criteria were defined *a priori*, before conducting the analysis, to ensure a degree of rigour and objectivity in our detection of threshold responses. It should be noted that the criteria were developed by ourselves, based on what we considered to be consistent with good practice. Different results may have been obtained had other criteria been adopted.

## Results

Over half (44/86) of the measured variables showed non-linear responses over the dieback gradient in this study, of which 13 exhibited thresholds according to our criteria. Here we identify the most clearly defined thresholds (i.e. those associated with small confidence intervals) pertaining to biodiversity, ecosystem function and ecological condition (see Supplementary Table S2 for additional results).

### Biodiversity

**Figure 2:**
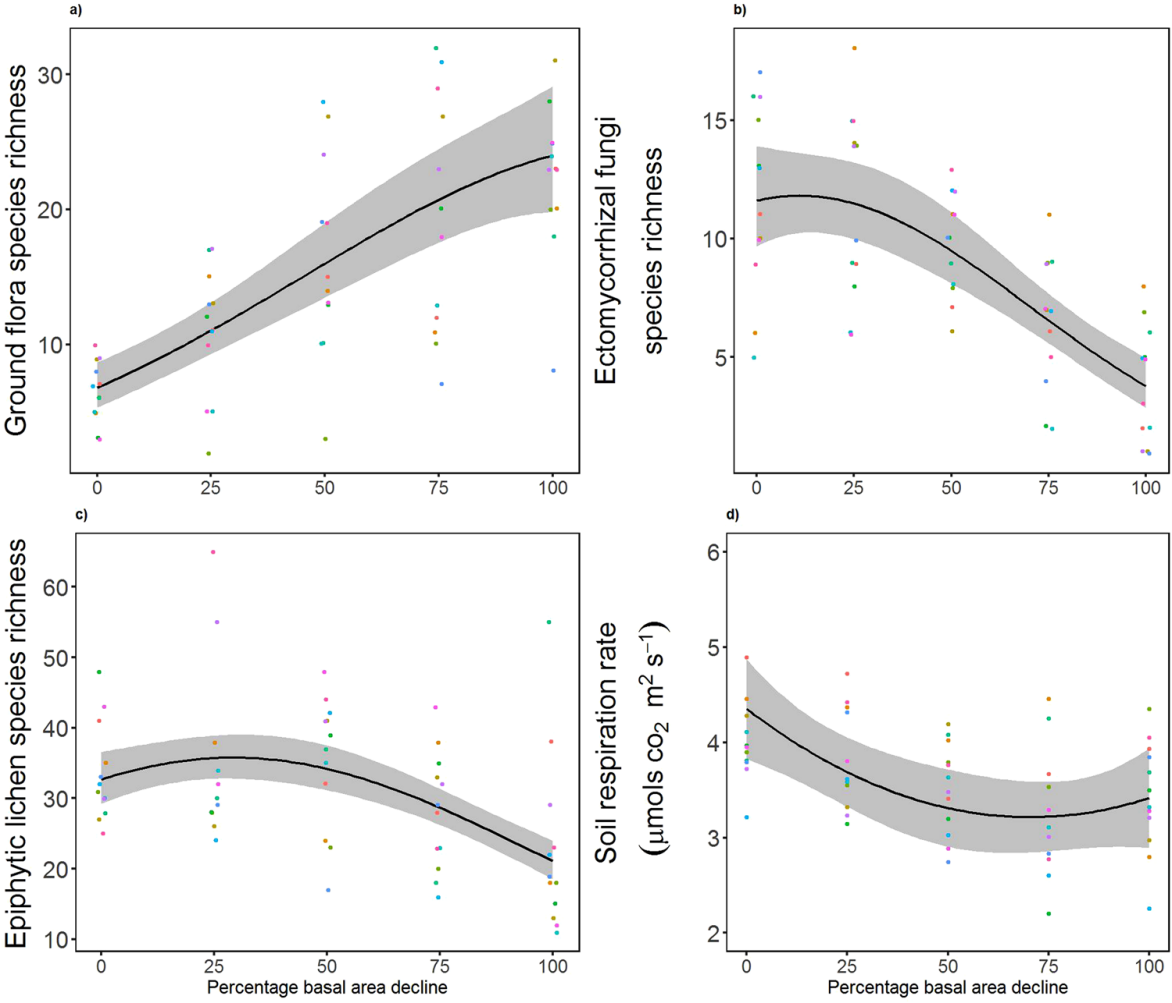
Threshold relationships between stage of dieback and species richness and soil respiration rate. Relationships between stage of dieback and species richness of (**a**) vascular ground flora (n = 60); (**b**) ectomycorrhizal fungi (n = 60); (**c**) epiphytic lichen (n = 60); and (**d**) soil respiration rate (n = 60). The black lines represent prediction using the most parsimonious model coefficients and grey shading the 95% confidence intervals of the coefficients (marginal *r* ^2^ = 0.60, 0.57, 0.44, and 0.16 for (**a–d**), respectively). The different coloured points represent the values at each individual site. All species richness values are the number of unique species found in 0.04 ha.

The relationship between ground flora species richness and dieback was best predicted by a regression model with a quadratic term of BA loss and a dung predictor term for all ground flora (*r* ^2^ = 0.60, ΔAICc = 5.37) (Fig. 2a) and for ground flora not including woody species (*r* ^2^ = 0.66, ΔAICc = 6.24). The most parsimonious ECM species richness model exhibited a threshold, with a quadratic term of BA loss (*r* ^2^ = 0.57, ΔAICc = 8.30) (Fig. 2b). In addition, total epiphytic lichen species richness exhibited a threshold response, with linear and quadratic terms of BA loss and a holly abundance term included in the most parsimonious model (*r* ^2^ = 0.44, ΔAICc = 19.1) (Fig. 2c), while lichen species richness on beech trees specifically also exhibited a threshold response (*r* ^2^ = 0.60, ΔAICc = 57.32), exhibited by having a quadratic and linear BA loss as its terms. Thresholds were not present in ground-dwelling arthropod richness, which was best represented by a linear BA term (*r* ^2^ = 0.26, ΔAICc = 2.41) (see Fig. S2a) or tree seedling richness, which was also best represented by a single linear BA term (*r* ^2^ = 0.19, ΔAICc = 2.02). Excluding the additional predictors of dung and holly abundance from ground flora and lichen analysis, respectively, all ground flora (*r* ^2^ = 0.55, ΔAICc = 8.00), ground flora not including woody species (*r* ^2^ = 0.61, ΔAICc = 15.62) and total epiphytic lichen species richness (*r* ^2^ = 0.24, ΔAICc = 12.20) were still best predicted by models with a quadratic term of BA loss, thus exhibiting thresholds (Supplementary Information, Table S4).

### Ecosystem functions

Only a single threshold response was exhibited in the 27 soil function variables measured over the dieback gradient, namely the case of soil respiration rate, which was demonstrated by quadratic term of BA loss included in the most parsimonious model (*r* ^2^ = 0.16; ΔAICc = 3.71) (Fig. 2d). For other soil functions, models with non-linear terms were often the most parsimonious models; however, these displayed very low *r* ^2^ and ΔAICc values and were not therefore considered to be exhibiting thresholds. These included potentially mineralisable nitrogen in the mineral layer (*r* ^2^ = 0.07; ΔAICc = 0.53) (PMNM; see Fig. S2b) and N mineralisation (*r* ^2^ = 0.13; ΔAICc = 0.97) (see Fig. S2c). Other modelled soil function results indicated that strong linear relationships were exhibited in the exchangeable cations of Na (*r* ^2^ = 0.34; ΔAICc = 7.06) and Ca (*r* ^2^ = 0.18; ΔAICc = 3.91). Total carbon storage was best predicted by a model with solely a linear BA term (*r* ^2^ = 0.50; ΔAICc = 1.14) (see Fig. S2d). The most parsimonious models for all other soil function variables either had lower *r* ^2^ values, or were best modelled by null models.

### Ecological condition

**Figure 3:**
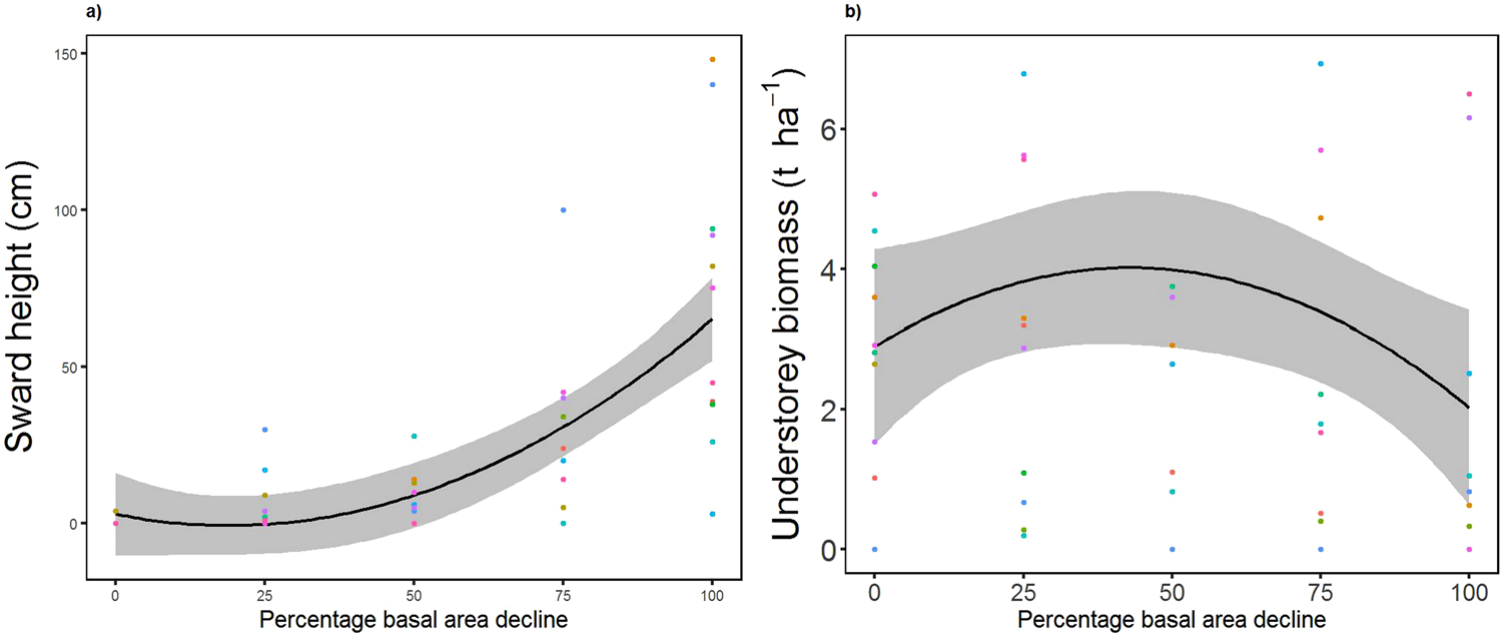
Threshold relationships between stage of dieback and ecosystem condition. Relationships between stage of dieback and (**a**) average sward height (n = 60); and (**b**) understorey biomass (n = 60). The black lines represent prediction using the most parsimonious model coefficients and grey shading the 95% confidence intervals of the coefficients (marginal *r* ^2^ = 0.51 and 0.38 for (**a**) and (**b**), respectively). The different coloured points represent the values at each individual site.

A threshold response in the average sward height was defined by the most parsimonious model having a quadratic term of BA loss (*r* ^2^ = 0.51; ΔAICc = 17.74) (Fig. 3a). Similarly, some of the seedling abundances (palatable seedlings, beech and oak separately and combined) showed thresholds effects, the most pronounced of which was the abundance of palatable seedlings, which had a quadratic term of BA loss and a dung factor (*r* ^2^ = 0.29; ΔAICc = 55.51). The understorey biomass also exhibited a threshold response as determined by the most parsimonious model, with a quadratic term for BA loss (*r* ^2^ = 0.38; ΔAICc = 5.81) (Fig. 3b). The condition of the remaining crowns was best described by a linear model, with only a linear BA loss term (*r* ^2^ = 0.16; ΔAICc = 2.22).

## Discussion

Our results provide novel evidence of thresholds in biodiversity, ecosystem function and ecological condition in a forest ecosystem undergoing dieback. The most striking threshold responses were observed for biodiversity, specifically in the species richness of ECM fungi and epiphytic lichens, both of which started to decline sharply with a decline in BA, and ground flora, which increased until the latter stages of the BA gradient. With respect to ecosystem function, a single threshold response was identified, namely in soil respiration rate. For ecological condition, thresholds were shown in sward height, which increased after initial decline in BA, and palatable seedling abundance, which initially increased across the gradient of stand BA, but started to decline in the late stages.

Previous research has reported a number of threshold responses in forest ecosystems as a result of deforestation and habitat fragmentation, including thresholds in forest structure^47, 48^, biodiversity loss^49, 50^ and ecosystem service provision^51^. Moreover, thresholds have been identified across forest-savanna-grassland gradients in tropical landscapes^52–54^. These studies all focused on the impacts of direct human-driven loss (i.e. physical removal) of forest cover or modified disturbance regimes at the landscape scale. As far as we are aware, the current study is the first to report threshold responses over a gradient of stand dieback, which represents a different form of ecosystem change than deforestation. Such dieback is increasing in response to environmental change in forests globally as a result of climate change, pest and disease attack, and increasing fire frequency^15, 55, 56^. Moreover, Allen *et al*.^57^ suggest that all forests may be vulnerable to climate-induced dieback in the future. The current results suggest that many other forest ecosystems that are being affected by dieback may potentially be characterised by threshold responses to environmental change.

The basis of ecological threshold theory is that rapid, non-linear changes are observed in ecosystem ‘state’ as a controlling variable changes^58^. This implies that a relatively small increase in intensity or frequency of disturbance could cause rapid and abrupt declines in ecosystem condition, state or function, potentially creating highly degraded ecosystems^59, 60^. This is concerning as thresholds may compromise the capacity of forest ecosystems to recover from future perturbations^61, 62^, especially as anthropogenic pressures are predicted to intensify in future^57, 63, 64^.

The precise mechanisms underlying ecological thresholds remain unclear^58^. Walkers and Meyers^65^ and Scheffer *et al*.^7^ have highlighted that in order for a threshold to occur there must be a switch in an ecosystem from a self-regulating state (negative feedback) to one that is reinforced by further internal or external changes (positive feedback), i.e. a self-exacerbating state^66^. The thresholds we observed in our study may be the result of a number of positive feedback mechanisms including interactions between trees, soil microbes, soil chemistry and herbivory. For example, as trees die and degrade, symbiotic associations with ECM fungi are reduced^67, 68^. This can cause reductions in the abundance of other soil microorganisms owing to major modifications to water and nutrient exchanges^69–71^, which could create a positive feedback that substantially lowers plant survival and growth^71, 72^. This could be evidenced by the decline in soil respiration rate that was observed in this study. In addition, the threshold observed in lichen species richness could be attributable to feedbacks between declining availability of bark substrate and changes in microclimate during the process of stand dieback^73, 74^.

In contrast to biodiversity measures, relatively little evidence was obtained here of threshold responses in measures of ecosystem function. In most cases, such measures varied non-linearly with BA decline, however, they did not fulfil the ΔAICc ≥ 3 and marginal *r* ^2^ > 0.15 criteria. The exception was soil respiration rate, which only narrowly exceeded the marginal *r* ^2^ criterion limit (*r* ^2^ = 0.15). As soil respiration is a net result of the respiration of autotrophic (plant) and heterotrophic (microbial and mycorrhizal) activity^75^, the initial declining trend may have been largely a result of decline in microbial activity in the soil owing to declining tree root density^76^ and tree presence^77^. In all cases, our interpretation of these data was based on the assumptions underlying space-for-time substitution, which should be borne in mind when interpreting the results.

The key assumption the approach adopted here, a space-for-time substitution, is that variation detected over space accurately reflects the ecological changes occurring over time. To reduce uncertainty in this study, this assumption was tested with results obtained from a long-term monitoring investigation in the same study area undertaken by Martin *et al*.^24^, which involved a beech woodland stand (Denny Wood) that has undergone stand dieback over the past 50 years. It showed that trends in BA depended on the scale at which the results were analysed; at the scale of 20 × 20 m plots (as employed in this study), BA decline was often strongly non-linear, displaying clear thresholds^24^. However, at the scale of the entire stand, BA decline was described by a linear trend. This reflects the fact that dieback of different parts of the stand was asynchronous. Of the stands that declined in BA in Denny Wood, mean values declined from 49 m^2^ ha^−1^ to 23 m^2^ ha^−1^ over a 50 year period^78^.

A further assumption of the space-for-time substitution approach is that all other conditions are the same across the plots surveyed^34^. Fukami and Wardle^79^ describe several ways to overcome this limitation. One is to include multiple sites, to uncover trends in ecosystem dynamics. In this study, 12 replicate sites were used to achieve this, with environmental condition measurements made pertaining to growing conditions and disturbance, two factors that influence woodland growth and mortality. Droughts and waterlogging events affect growth and mortality of beech^80–82^, especially in southern England^30, 83^, with the clay content of soil affecting how quickly water drains away. Particle size distribution analysis of soil samples from all sites demonstrated that the percent clay soil content did not change significantly (F (4,55) = 0.177, P = 0.949) (Supplementary Information, Fig. S3a) across the dieback gradient, based on one-way ANOVA results. This indicated that drought or waterlogging could have had the same effect on any plots across the dieback gradient. Other variables that could have identified the stands as having different conditions, or being of different ages all also had no significant variation over the gradient: organic soil depth (F (4,55) = 1.160, P = 0.338) (Supplementary Information, Fig. S3b), which suggests that similar values of soil moisture, organic nutrients and stability were present among sites; soil pH (F (4,55) = 0.910, P = 0.465) (Supplementary Information, Fig. S3c), which indicates that all the stands were similarly acidic and therefore are characterised by similar processes such as nutrient uptake that are dependent on pH; and dbh of the remaining living trees (*x* ^*2*^ (3) = 0.586, P = 0.899) (Supplementary Information, Fig. S3d), which indicates that trees were of a similar age and grew in similar conditions, based on the result of a Kruskal-Wallis test. Overall, the assumption that environmental conditions were comparable across the gradients was supported by these data. Furthermore, no significant differences across the gradient were exhibited in the measures of herbivore dung (*x* ^*2*^ (4) = 1.866, P = 0.760) (Supplementary Information, Fig. S4a) and the percentage of holly stand bases that were browsed (F (4,55) = 1.386, P = 0.251) (Supplementary Information, Fig. S4b), indicating that herbivore pressure was uniform across the dieback gradient.

There were a few other issues relating to data interpretation which should be borne in mind when interpreting the results. First, in near-natural beech forests, the mortality of overstorey trees and regeneration are typically synchronized within a period of several decades, in patches extending over several hectares^84^. The beechwoods of the New Forest differ from this situation, however, owing to the very high browsing pressure from large herbivores^24^. As a result, beech regeneration is very sparse, and dieback of woodland stands often involves conversion to non-woodland habitat, principally grassland^24^. Second, mortality processes in trees are often highly complex and difficult to interpret^85^. This complexity is illustrated by other studies of stand dieback in tree species. For example in studies of sudden dieback of aspen stands in North America, a number of different contributory and potentially interacting factors were identified, including drought, defoliation, extreme weather events and wildlife stem damage^86^. Similarly in their review of drought impacts on temperate forest stands, Bréda *et al*.^87^ identify a number of different physiological mechanisms that can increase the risk of tree mortality following drought, including decreased carbon and nutrient assimilation, breakdown of the photosynthetic machinery, and reduced storage of carbohydrates. In the New Forest, causes of large beech mortality has previously been attributed to drought, with increasing frequency of droughts resulting in numerous serious water deficits since 1976, although the evidence for this is uncertain^24^. Additional mortality factors could include significant storms that occurred in 1987 and 1990 and fungal pathogens attacks, which have been observed affecting beech the New Forest^24^. Moreover, while factors such as insect attack, frost damage and bark stripping by herbivores were not analysed here, they could have had a significant impact on mortality patterns at this site. It should also be noted that the causes of the dieback observed could also potentially be correlated with the response variables; for example, increased incidence of drought could have concurrently affected both the survival of individual trees and the ECM fungi with which they are associated.

Further, it should be noted that data were evaluated from a single sample period along a gradient of live-tree BA. Ideally, data would have been obtained by sampling the same plots before and after the initiation of tree dieback. As noted above, the only long-term data available for this study relate to one of the 12 sites surveyed, namely Denny Wood^24^. Our interpretation of the results is therefore based on the assumption that the sequential dieback of beech that has been documented at that site also applies to the other sites in the New Forest where BA gradients were surveyed. In addition, it is important to note that we interpret here differences in the ecosystem composition, structure, and function among the plots as a response to dieback. It is conceivable that the variables measured could have differed across the study area prior to the onset of dieback. For instance, soil respiration might have varied across the study area prior to the onset of dieback, and this could have contributed to some of the variation in the magnitude of dieback observed. We have no way of testing whether all of the variables measured differed between measurement locations prior to the onset of dieback, and therefore our attribution of the responses observed to dieback is based on an assumption that there was no systematic variation in these variables prior to the occurrence of dieback.

Other issues that have a bearing on the interpretation of our results include our definitions of a threshold and dieback. Here we considered a response variable to show a threshold if it met the three criteria described in the Methods. As the criteria were developed by ourselves, different results may have been obtained had other criteria been adopted. Moreover, the definition of dieback we adopted was a decline in stand BA as the central measure. This is based on the results of a review of previous research conducted by^33^, into the forest ecosystem characteristics that have most often found to be significantly related to maintenance of forest biodiversity. Of these, BA is one of the forest stand structure variables most consistently associated with forest biodiversity and with aspects of the functioning of forest ecosystems, such as carbon storage^33^.

## Conclusion and Implications

Climate-induced forest stand dieback is rapidly increasing worldwide, in scale, magnitude, severity and speed^57^. The occurrence of thresholds in forest ecosystems undergoing dieback is a major concern, since continued environmental change may produce non-linear declines in biodiversity and ecosystem function as the result of linear changes in disturbance. Our results indicate that such thresholds can occur over a BA gradient in a forest undergoing dieback. Importantly, our results show that species richness of ECM and epiphytic lichen start to decline sharply before there is a 50% decline in BA, which implies a shift from negative feedback mechanisms to strong positive feedbacks at this threshold. In contrast, only one ecosystem function measured, namely soil respiration rate, displayed a threshold response, suggesting that biodiversity and ecosystem function threshold responses are not necessarily closely coupled. Further research is required to identify the precise mechanisms underlying the threshold responses observed, and to examine whether the observed changes are reversible.

## Electronic supplementary material

Supplementary Information
