# Supplementary material for: Thresholds of biodiversity and ecosystem function in a forest ecosystem undergoing dieback
Source: Sci Rep. 2017 Jul 28;7:6775. doi: 10.1038/s41598-017-06082-6 (PMC5533776; doi:10.1038/s41598-017-06082-6)
Supplement: Supplementary file 1 — Supplementary Information [file 41598_2017_6082_MOESM1_ESM.pdf]

Supplementary Information

for

‘Thresholds of biodiversity and ecosystem function in a forest ecosystem undergoing dieback’

P. M. Evans[1], A. C. Newton[1], E. Cantarello[1], P. Martin[1], N. Sanderson[2], D. L. Jones[3], N. Barsoum[4], J. E. Cottrell[4], S. W. A'Hara[4], L. Fuller[5]

[1]Centre for Conservation Ecology and Environmental Sciences, Faculty of Science and Technology, Bournemouth University, Poole, BH12 5BB, UK.

[2]Botanical Survey and Assessment, 3 Green Close, Woodlands, Southampton, Hampshire, SO40 7HU, UK.

[3]School of Environment, Natural Resources and Geography, Bangor University, Gwynedd, LL57 2UW, UK.

[4]Forest Research, Alice Holt Lodge, Farnham, Surrey, GU10 4LH, UK.

[5]Biological and Environmental Sciences, University of Stirling, Stirling, FK9 4LA, UK.

Tel +44 (0) 1202 961831

pevans@bournemouth.ac.uk

Inventory of Supplementary Information:

Table S1: Basal area statistics.

Fig. S1: Graph of stand basal area over the dieback gradient**.**

Fig. S2: Non-threshold relationships between stage of dieback and ecosystem processes.

Supplementary Methods, SM1: Additional methods for experimental design and most data collection.

Supplementary Methods, SM2: Methods for collecting and analysing ground-dwelling arthropods data.

Table S2: Summary of variables measured and units used.

Table S3: Generalised linear mixed models used for the study and their results.

Table S4: Updated version of Table S3 with only linear and quadratic term of BA included as fixed effects.

Table S5: Statistics of the soil properties.

Supplementary Methods, SM3: Graphs to support the space-for-time assumption.

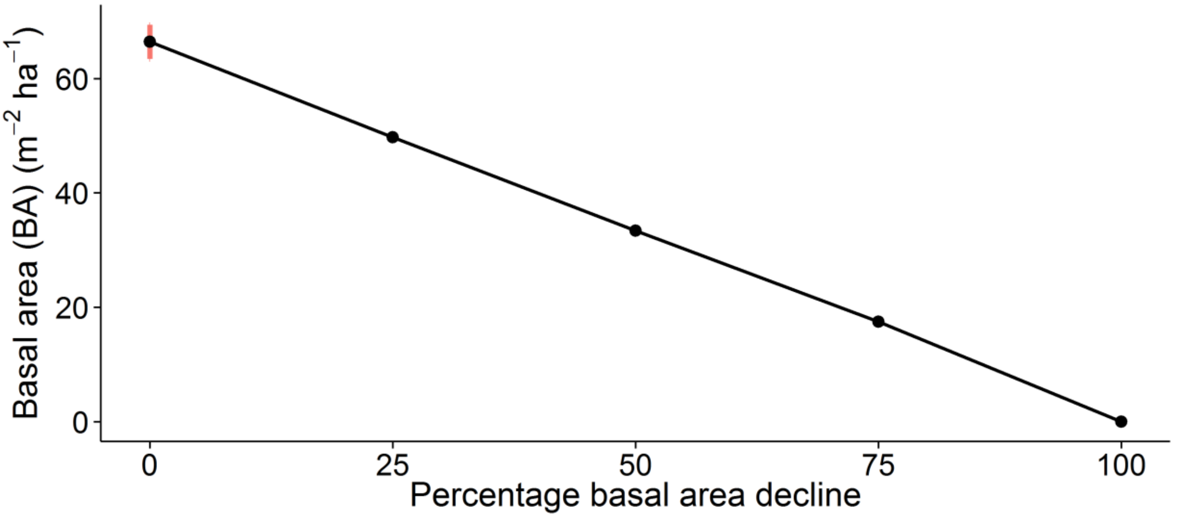

Fig. S1: The mean stand basal area (BA) of dieback stages of the gradient plots. Standard error bars are shown in red.

		BA					
**Percent basal area decline**	**N**	**Mean**	**SD**	**SE**	**CI**	**Min**	**Max**
0%	12	66.42	10.29	2.97	6.54	59.85	98.39
25%	12	49.71	1.36	0.39	0.86	47.73	52.12
50%	12	33.37	1.79	0.52	1.14	30.58	37.12
75%	12	17.45	1.47	0.42	0.93	13.65	19.44
100%	12	0	0	0	0	0	0

Table S1: Basal area (BA) statistics. Mean, standard deviation (SD), standard error (SE), confidence interval (CI), minimum (Min) size of BA and maximum (Max) size of BA for each of the stages of dieback.

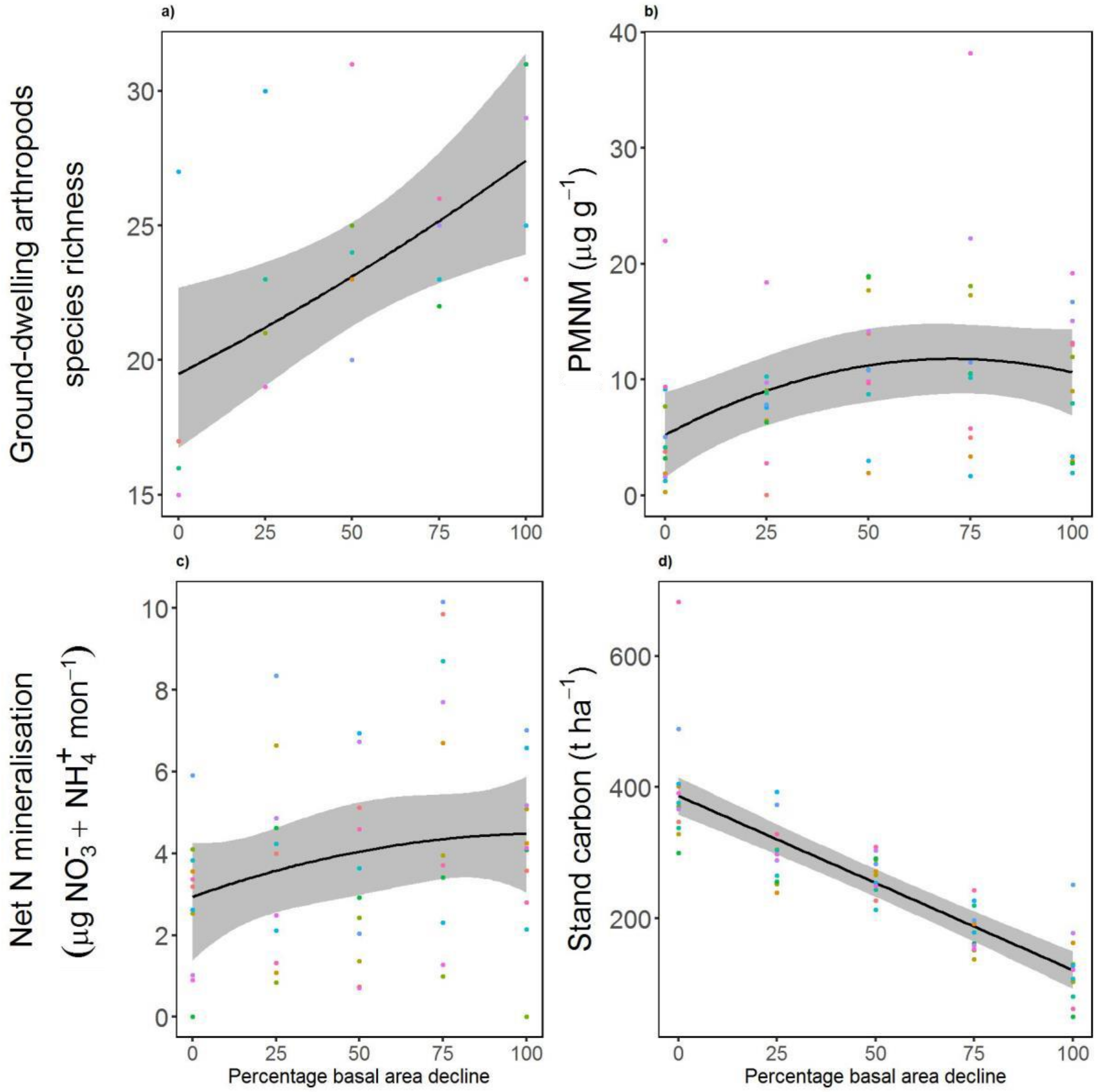

Fig. S2: Non-threshold relationships between stage of dieback and ecosystem processes. Relationships between stage of dieback and a) ground-dwelling arthropods (n = 25); b) potentially mineralisable nitrogen in the mineral layer (PMNM) (n = 60); c) net mineralisation per month (n = 55); and d) total stand carbon (n = 60). The black lines represent prediction using the most parsimonious model coefficients and grey shading the 95% confidence intervals of the coefficients (marginal r^2 =0.26, 0.07, 0.13, and 0.50 for a-d, respectively). Net mineralisation was measured as the amount of NH_4^+ and NO_3^- taken up by a resin capsule over a four month period and then divided by 4 to obtain a value per month. The different coloured points represent the values at each individual site.

Supplementary Methods: SM1

Plot set-up

Each plot was 20 x 20 m (400 m^2; 0.04 ha). The edges were delineated with measuring tapes. A compass was used to confirm that the adjacent angles were at 90˚ angles. A nested sub-plot of 10 x 10 m (100 m^2) was set up in the centre of each plot, laid out in the same orientation as the full plot. The centre and the corners of the sub-plot were marked with wooden stakes for easy identification on return visits. The mid-points of each plot were recorded using a handheld GPS (GPSMAP 60CSx; Garmin, USA).

Structural survey

The diameters at breast height (dbh) of both living and dead standing trees (snags) were measured at 1.3 m using a diameter tape pulled taut horizontally to the trunk. Following advice and procedures from Husch *et al.*[1] and van Laar and Akça[2], specific instructions were followed when using diameter tapes for difficult trees. The combined dbhs were used to calculate the overall BA [3], forming the basis of the primary criterion.

Crown condition

Living beech trees were further assessed for their condition, undertaken using binoculars at several points around each tree where visibility was good. The condition attributes were the potential crown loss, live growth loss, condition of the current branches and discolouration of the crown. Potential crown loss and leave loss were recorded as a percentage based on the average values provided by two observers. Similarly, condition was recorded as number (1-4) based on the descriptions. Any pathogens present were also recorded after a thorough search of the lower sections of each tree.

Canopy openness

At each corner of the 10 x 10 m sub-plot four readings were taken using a spherical densiometer, one in each cardinal direction, giving an overall average for that plot[4].

Understorey openness

Understorey openness was determined the same way as canopy openness, but only for trees less than approximately 6 m in height.

Forest biomass

Following Jenkins *et al.*[5], oven-dry biomass was determined in four different components of the stand; the roots, the tree stems, the branches and foliage. To calculate the total biomass of a single species, the stem biomass, crown biomass and root biomass were summed together and multiplied by the number of that species present in the plot. The total biomass of all species was then calculated by summating all individual species' biomass values. The oven-dry biomass was calculated based on specific values for broadleaves, taken from McKay *et al*[6].

Carbon assessment for trees

Carbon content of a plot was calculated by multiplying the oven-dry matter biomass by 0.5, the carbon fraction of biomass[7].

Herbivore pressure metrics

To account for the relative presence and influence of herbivores, understorey crown condition, browseline, sward height, seedling and sapling abundance, browsing intensity, dung counts, and presence of a shrub layer were recorded.

For living trees in the understorey, crown condition (average of two different observers) was recorded based on deviation from perceived 'pristine' condition (i.e. 100%). Percentage of discolouration, percentage of leaves remaining, potential crown structure, empty branches and position of the tree were taken into account.

The browse lines of palatable (e.g. beech, oak, birch) and unpalatable (e.g. holly, hawthorn) trees were recorded if they were within the edges of the plot. Using a marked range pole, any branches that were higher than 1.8 m (a deer's maximum browse height), but lower than 2.3 m (based on an average drop of 50 cm in the winter), were counted as browsed. Any branches that retained leaves below 1.8 m were counted as unbrowsed. A percentage ratio of browsed to unbrowsed was calculated. The sward height was measured using a measuring stick, based on the findings of Stewart *et al.*[8] This was measured in the centre and at the four corners of the sub-plot, and a mean value was recorded.

The percentages cover of mosses, bare ground, bracken, trampling and ground flora were recorded from a detailed visual assessment of each plot. Similarly, seedling (< 1.3 m in

height) and sapling (> 1.3 m and dbh < 10 cm) abundances were assessed through a manual search of the entire 20 x 20 m plot. Seedlings were any counted if they were older than a year, based on physical aspects.

Partial defoliation or complete consumption of plants occur through herbivore browsing, the intensity of which is commonly determined by counts of un-browsed and browsed branches[9,10]. This was undertaken using a random stratified design. Initially, a 2 x 2 m quadrat was placed in the most south-westerly corner of the sub-plot, continuing clockwise (NW, NE, SE) around the corners, until 100 stems had been assessed. The same technique was used for assessing bramble browsing, following Bazely *et al* [11].

For estimating herbivore abundance from dung, the faecal standing crop (FSC) method, the most commonly used and efficient technique[12,13], was used. A manual dung count was carried out in the sub-plot; the amount, condition and the species recorded. Following Jenkins and Manly[14], the individual pellets/ bolus and their condition were recorded. The faecal matter of different animals (deer, *Equus* species, rabbits and cattle) were recorded separately.

Soil survey

Following the methods of DeLuca *et al.*[15], ten separate soil samples were taken in randomly-stratified positions, two from the centre and two at each corner of the nested 10 x 10 m sub-plot, for both the O horizon and A horizon soil layer (0-15 cm below the O horizon). The vegetation the sample was taken under (e.g. bracken, grass) was noted.

For bulk density (BD) measurements, three 100 cm^3 stainless steel rings were inserted into the soil to ensure a known volume. These were taken from the SW and NE corners and from the mid-point.

For analyses of NO_3^- and NH_4^+, 5 g of sieved, field-moist soil was placed into a labelled tube with 25 ml of 1 M KCl added. The soils were shaken by hand and placed horizontally on a rotary shaker for 30 minutes at 250 rev/min. The extracts were immediately filtered through a Fisher QT 210 filter paper into a labelled polypropylene vial. The filtrates were then frozen immediately and analysed two months later. Both NH_4^+ and NO_3^- were analysed using the microplate-colorimetric technique, with the salicylate-nitroprusside method for NH_4^+, following Mulvaney[16] and the vanadium method for NO_3^-[17].

To determine the potential mineralisable nitrogen concentrations, 5 g of sieved, field-moist soil was placed into a labelled tube with 25 ml of ultrapure water added. The headspace was then flushed with N_2 (g). The tube was sealed and incubated for 7 days at 40°C [18]. Immediately after incubation, 1.75 g of KCl was added to each tube. The tubes were shaken (1 hr at 200 rev/min), centrifuged and filtered immediately, using the process as for NO_3^- and NH_4^+. The pH and electrical conductivity of soil was determined using a 2:1 deionized water to soil ratio.

Net N mineralisation and nitrification:

To enable analysis of in-situ of nitrification and N mineralisation rates, following DeLuca *et al.*[15], a polyester mesh ionic resin capsule (Unibest, Walla Walla, WA, USA) was buried in the centre of each plot, 10 cm deep into the mineral layer. The capsules were placed between 9th October and 12th November, 2014 and were removed from the ground four months later.

The nitrogen mineralisation and nitrification of a plot were analysed through leaching of resin capsules (RC). Initially, 10 mL of 1 M KCl was placed into each tube containing a RC, which was then shaken horizontally for 30 minutes at 250 rpm. The extractant was poured into a clean storage tube. This process was repeated two more times, making a total of 30 mL of the extractant. The extractant was centrifuged at 4000 rpm for 10 minutes. 20 mL of the supernatant was then pipetted into a 30 mL polypropylene tube and frozen prior to colorimetric analysis as described above.

Soil respiration rate:

Soil respiration rate was measured using a SR-1 closed chamber Infra-red gas analyser (PP Systems, Amesbury, MA, USA). All measurements were recorded between 10:00 and 14:00 on sunny days within a month of each other. After automatic flushing and calibration of the chamber, the PVC chamber was inserted 2 cm into the soil after any vegetation had been removed from the surface. The CO_2 concentration was measured continuously for 2 minutes. Five measurements were taken from each survey plot and then averaged to produce a mean soil respiration rate for the whole plot. Soil respiration rate was calculated as in (PP Systems[19]:

$$R = V/A \times ((Cn - Co)/(Tn))$$

Where *R* is the respiration rate, *V* is the volume of the chamber, *A* is the area of soil exposed, *Cn* is the CO_2 concentration at time 0, and *Co* is the CO2 concentration at time, *Tn* (120 seconds in this study).

Soil moisture

Soil moisture was measured as the difference in weight of a 5 g moist soil sample before and after oven-drying. Sieved mineral and organic samples were oven-dried at 105 °C and 80 °C, respectively, until they remained a constant weight. To measure the soil organic matter (SOM), the oven-dried samples were then placed in a 500 °C furnace overnight (12 hours), the final weight recorded after being cooled in a desiccator. LOI = 100 x (mass of oven-dry soil-mass of ignited soil)/ mass of oven-dry soil = g per 100g oven-dry soil[20]. The soil was dried at 105 °C for 24 h and then sieved (2 mm) to remove stones and other non-soil material (>2 mm diameter). Bulk density was calculated by dividing soil mass (less stone mass) by core volume (less stone volume).

Soil content and structure

The Forest Research (FR) team at Alice Holt Lodge, Surrey, measured the exchangeable cations/anions of K, S, Ca, Mg, Na, Al, Mn and F; total N and C, organic and inorganic C; the plant-available P; and the particle sizes of the soil from air-dried samples. Following FR methods, the exchangeable cations/anions were analysed using $BaCl_2$ extraction (FR Reference method: ISO 11260 & 14254). First, a soil suspension of 3 g soil and 36 ml of 0.1 M $BaCl_2$ was shaken for 60 minutes, centrifuged and filtered with 0.45 μm syringe filter. Extracts were then acidified and analysed using a dual view ICP-OES (Thermo ICap 6500 duo). The Olsen P method with ADAS index was used to determine the amount of phosphorus available (FR Reference method: The analysis of Agricultural Materials MAFF 3rd Edition RB427). A suspension of 5 g soil with 100 ml of sodium bicarbonate solution was buffered at pH 8.5. The solution was shaken for 30 min on an orbital shaker, centrifuged and filtered with 0.45μm syringe filters. Extracts were then acidified with 1.5 M sulphuric acid and mixed with a solution of ascorbic acid and ammonium molybdate for 10 min and then measured at 880 nm with a Shimadzu UV sprectrophotometer. Total C and N were analysed using a Carlo Erba CN analyser (Flash1112 series) and combustion method (FR Reference method: ISO 10694 & 13878). Samples were ball-milled for homogenisation and then around 30 mg weighed in tin capsules, pressed and measured using the analyser.

Following, 30 g of soil was placed in a silver capsule to quantify inorganic C. The silver capsule was put furnace at 500°C for 2 hours, which removed the organic carbon. The organic carbon fraction was calculated as the difference between total carbon and inorganic carbon. The soil particle size distribution was determined using a Laser Diffraction Particle Sizer (FR Reference method: Laser diffraction); 30 g of soil were suspended in water and passed through the flow cell of the analyser (Beckman Coulter LS13320).

Data analysis

Random intercepts and slopes were included for each site. All the variables were tested for normal distribution with the Shapiro–Wilk test and for homogeneity of variances for Bartlett's test[21]. Data that did not fit these assumptions were log-transformed prior to analysis.

Count data were modelled using a Poisson error structure. For proportional and percentage data, a small non-zero value was added to avoid infinite logit transformed values[22]. AICc values were calculated using the maximum likelihood value of the model[23]. AICc values were determined using the MuMIn R package[24] and used to define the most parsimonious model, following an information theoretic approach[23]. Performance of models was evaluated by calculating the marginal r^2 [25].

Supplementary Methods: SM2

Ground-dwelling arthropods collection

Pitfall trapping was carried out in five out of the 12 sites. In each site eight pitfall traps were placed on the perimeter of the 10m x 10m sub-plot; one in each corner and one midway along each edge. A soil auger was used to create holes in which plastic cups (8 cm in diameter and 11 cm tall) were placed. Approximately 3 cm of propylene glycol, a cost effective preservative, was poured into each cup. Water was allowed to escape through the use of drainage holes in the top of the cups; this also prevented the trap flooding. A galvanised steel square which was supported by turned-down corners was placed over each trap. Forestry Commission staff collected the contents of each pitfall trap weekly from late May to late July 2014, totalling eight collections and 56 trapping days. The arthropod material from the eight pitfall traps in each plot were pooled into a single labelled and sterilised 1 litre sample bottle and then stored in -5 °C to preserve the specimens for metabarcoding.

Ground-dwelling arthropods analysis

DNA metabarcoding was employed for invertebrate identification using a methodology tailored from the approach described in Yu *et al.*[26]. Samples were stored in absolute ethanol at 4°C, followed by the extraction of DNA using the Qiagen blood and tissue extraction kit. Polymerase Chain Reactions (PCR) were performed targeting the 658 base pair C terminal region in the gene encoding the mitochondrial cytochrome oxidase subunit I (COI); primers used for the COI region of interest were: Forward: LCO1490 (5'-GGTCAACAAATCATAAAGATATTGG-3') and Reverse: mlCOIintGLR (5'-GGNGGR TANANNGTYCANCCNGYNCC-3'). Three separate PCRs were carried out for each sample. An aliquot was checked on a 1.4% agarose gel and then the PCRs pooled before library construction. A multiplex identifier (MID) tag was attached to the forward primer in addition to the relevant adaptor for the sequencing platform. The MID tag was specific to each sample and allowed multiple samples to be pooled for sequencing and then separated out bioinformatically afterwards. A touch-down thermocycling profile was used, followed by a low number of cycles with an intermediate annealing temperature. Indexing barcodes were added to the amplicons following the Illumina TruSeq Nano protocol from the 'Clean-up Fragmented DNA' stage. In a deviation from this protocol fragments were size-selected using blue Pippin size selection of the 300-670bp region to remove larger fragments. The barcoded samples were pooled into a single pool and 250bp paired end reads were generated on one

lane of the Illumina MiSeq platform. The pool was demultiplexed into the individual samples using the Illumina bcl2fastq (v 1.8.4bin) software. The samples were clustered into OTUs (operational taxonomic units) using the approach described in Yu *et al.*[27] starting with demultiplexed samples in step 1. Instead of the described step 6 of the pipeline we used the BOLD database and website for taxonomic assignment and confidence assessment. Accepted matches had to have at least 97% sequence similarity at a given taxonomic level. For this we queried the website by using a custom script that created the urls and parsed the output for each OTU. In a final step the taxonomic assignment, OTU and the number of reads of each sample mapping to the OTUs was collated into a single table. The final species lists were checked against previous records of species occurrence in Britain using primarily the National Biodiversity Networks Gateway[27] but also Fauna Europapaea[28], Antweb[29], the British Arachnological Society[30,] and Araneae: Spiders of Europe[31]. Where no previous record was found to species level, occurrence in Britain to Genus level was checked.

Table S2: Summary of variables measured and units used.

Variable	Biodiversity (B), ecosystem function (EF) or ecosystem condition (EC) measure?	Units
Ectomycorrhizal fungi species richness	B	Unique species 0.04 ha^{-1}
Sward height	EC	cm
Abundance of holly seedlings	EC	Individuals 0.04 ha^{-1}
Abundance of beech seedlings	EC	Individuals 0.04 ha^{-1}
Abundance of oak seedlings	EC	Individuals 0.04 ha^{-1}
Abundance of tree seedlings	EC	Individuals 0.04 ha^{-1}
Abundance of palatable seedlings	EC	Individuals 0.04 ha^{-1}
Bulk density of the soil	EC	g cm^{-3}
Depth of the organic layer	EC	cm
Average diameter at breast height of beech trees	EC	cm
Average height of beech trees	EC	m
Volume of standing deadwood in a plot	EC	m^3 ha^{-1}
Volume of lying deadwood in a plot	EC	m^3 ha^{-1}
C/N ratio of the soil	EF	C/N ratio
Potassium exchangeable cations concentration in the mineral layer soil	EF	cmol(+)/kg
Magnesium exchangeable cations concentration in the mineral layer soil	EF	cmol(+)/kg
Sodium exchangeable cations concentration in the mineral layer soil	EF	cmol(+)/kg
Calcium exchangeable cations concentration in the mineral layer soil	EF	cmol(+)/kg
Manganese exchangeable cations concentration in the mineral layer soil	EF	cmol(+)/kg
Iron exchangeable cations concentration in the mineral layer soil	EF	cmol(+)/kg
Aluminium exchangeable cations concentration in the mineral layer soil	EF	cmol(+)/kg
Availability of soil phosphorus	EF	mg kg^{-1}
Total soil nitrogen	EF	% of soil
Total soil carbon	EF	% of soil
Soil pH	EF	pH
Electrical conductivity	EF	mS m^{-1}
Net ammonification	EF	µg NH_4^+ $capsule^{-1}$ mon^{-1}
Net nitrification	EF	µg NO_3^- $capsule^{-1}$ mon^{-1}
Net mineralisation	EF	µg NH_4^+ and NO_3^- $capsule^{-1}$ mon^{-1}
Soil respiration rate	EF	µmol m^{-2} s^{-1}

Soil temperature	EF	°C
Total stand carbon (vegetation, deadwood and soil)	EF	t ha^{-1}
Aboveground biomass	EC	t ha^{-1}
Soil clay percentage	EC	% 0-2 µm soil particles
Soil silt percentage	EC	% 2-63 µm soil particles
Soil sand percentage	EC	% 63 µm-2 mm soil particles
Bracken cover	EC	% cover 0.04 ha^{-1}
Bare ground and moss cover	EC	% cover 0.04 ha^{-1}
Litter cover	EC	% cover 0.04 ha^{-1}
Grass cover	EC	% cover 0.04 ha^{-1}
Palatable tree browseline	EC	% browseline (above 1.8 m) 0.04 ha^{-1}
Unpalatable tree browseline	EC	% browseline (above 1.8 m) 0.04 ha^{-1}
Holly cover	EC	% cover 0.04 ha^{-1}
Rubus cover	EC	% cover 0.04 ha^{-1}
Holly shrubs browsed	EC	% browse of available plants
Rubus shrubs browsed	EC	% browse of available plants
Average crown condition	EC	% condition
Understorey condition	EC	% condition
Canopy openness	EC	% sky visible
Understorey openness	EC	% sky visible
Tree seedling richness	B	Unique species 0.04 ha^{-1}
Tree sapling richness	B	Unique species 0.04 ha^{-1}
Spider species richness	B	Unique species 0.04 ha^{-1}
Rove beetles species richness	B	Unique species 0.04 ha^{-1}
Carabid beetles species richness	B	Unique species 0.04 ha^{-1}
Ant species richness	B	Unique species 0.04 ha^{-1}
Weevil species richness	B	Unique species 0.04 ha^{-1}
Woodlice species richness	B	Unique species 0.04 ha^{-1}
Ground-dwelling arthropod species richness	B	Unique species 0.04 ha^{-1}
Moisture content of the mineral layer	EF	% soil moisture
Moisture content of the organic layer	EF	% soil moisture
Cervus dung proportional	EC	see Jenkins and Manly (2008)
Equus dung proportional	EC	see Jenkins and Manly (2008)
Proportional dung total	EC	see Jenkins and Manly (2008)
Very large beech trees (74.97 cm < dbh < 103 cm)	EC	Individuals 0.04 ha^{-1}
Large beech trees (68.32 cm < dbh < 74.97 cm)	EC	Individuals 0.04 ha^{-1}

Holly tree abundance	EC	Individuals 0.04 ha^{-1}
Beech trees abundance	EC	Individuals 0.04 ha^{-1}
Holly saplings abundance	EC	Individuals 0.04 ha^{-1}
Beech saplings abundance	EC	Individuals 0.04 ha^{-1}
Overall saplings abundance	EC	Individuals 0.04 ha^{-1}
Ground flora species richness	B	Unique species 0.04 ha^{-1}
Woody ground flora species richness	B	Unique species 0.04 ha^{-1}
Non-woody ground flora species richness	B	Unique species 0.04 ha^{-1}
Lichen species richness	B	Unique species 0.04 ha^{-1}
Lichen species richness on holly	B	Unique species 0.04 ha^{-1}
Lichen species richness on beech	B	Unique species 0.04 ha^{-1}
Organic layer loss on ignition	EC	% weight loss
Mineral layer loss on ignition	EC	% weight loss
Organic layer nitrate concentration	EF	mg kg^{-1}
Mineral layer nitrate concentration	EF	mg kg^{-1}
Organic layer ammonium concentration	EF	mg kg^{-1}
Mineral layer ammonium concentration	EF	mg kg^{-1}
Potentially mineralisable nitrogen of the organic layer	EF	µg g^{-1}
Potentially mineralisable nitrogen of the mineral layer	EF	µg g^{-1}
Understorey biomass	EC	t ha^{-1}

Table S3: Generalised linear mixed models used to determine whether a threshold was exhibited in all the response variables and associated measures of parsimony (AICc), support (ΔAICc, AICc weight) and goodness of fit (Marginal r2). Mod_cont_NL specifies that the model contained a linear and quadratic term of BA loss indicating a non-linear response; Mod_cont specifies that the model only contained a linear term of BA loss indicating a linear response; and Modnull1 specifies that the model indicated little or no change over the gradient of BA loss.

Response variable	Name	Model structure	df	Log likelihood	AICc	ΔAICc	AICc weight	Marginal r^2	Threshold?
**Ectomycorrhizal fungi species richness**	Mod_cont_NL	BA decline + BA $decline^2$	4	-149.400	307.526	0.000	0.984	0.568	Yes
	Mod_cont	BA decline	3	-154.700	315.824	8.298	0.016	0.463	
	Modnull1	Null model	2	-185.130	374.476	66.949	0.000	0.000	
**Sward height**	Mod_cont_NL	BA decline + BA $decline^2$	5	-264.500	540.106	0.000	1.000	0.507	Yes
	Mod_cont	BA decline	4	-274.560	557.849	17.743	0.000	0.416	
	Modnull1	Null model	3	-294.110	594.648	54.542	0.000	0.000	
**Abundance of holly seedlings**	Mod_cont_NL	BA decline + BA $decline^2$ +log(Dung)	5	-1332.800	2676.800	0.000	1.000	0.119	No
	Mod_cont	BA decline +log(Dung)	4	-1844.600	3697.830	1021.030	0.000	0.047	
	Modnull1	Null model	3	-1891.800	3790.040	1113.250	0.000	0.007	
**Abundance of beech seedlings**	Mod_cont_NL	BA decline + BA $decline^2$ + log(Dung)	5	-275.660	562.439	0.000	1.000	0.216	Yes
	Mod_cont	BA decline + log(Dung)	4	-297.960	604.637	42.198	0.000	0.169	
	Modnull1	Null model	3	-331.090	668.610	106.172	0.000	0.015	

**Abundance of oak seedlings**	Mod_cont_NL	BA decline+ BA decline^2 + log(Dung)	5	-50.194	111.499	0.000	0.998	0.455	Yes
	Mod_cont	BA decline + log(Dung)	4	-57.726	124.178	12.679	0.002	0.176	
	Modnull1	Null model	3	-62.773	131.974	20.474	0.000	0.035	
**Abundance of tree seedlings**	Mod_cont_NL	BA decline+ BA decline^2 + log(Dung)	5	-1372.800	2756.790	0.000	1.000	0.134	No
	Mod_cont	BA decline + log(Dung)	4	-1902.900	3814.570	1057.780	0.000	0.051	
	Modnull1	Null model	3	-1967.100	3940.640	1183.850	0.000	0.001	
**Abundance of palatable seedlings**	Mod_cont_NL	BA decline + BA decline^2 + log(Dung)	5	-265.390	541.900	0.000	1.000	0.294	Yes
	Mod_cont	BA decline + log(Dung)	4	-294.340	597.407	55.507	0.000	0.226	
	Modnull1	Null model	3	-332.490	671.411	129.511	0.000	0.004	
**Bulk density of the soil**	Modnull1	Null model	3	17.940	-29.452	0.000	0.828	0.000	No
	Mod_cont	BA decline	4	17.350	-25.973	3.479	0.145	0.033	
	Mod_cont_NL	BA decline + BA decline^2	5	16.840	-22.568	6.883	0.027	0.038	
**Depth of the organic layer**	Modnull1	Null model	3	-26.750	59.929	0.000	0.740	0.000	No
	Mod_cont	BA decline	4	-27.262	63.251	3.322	0.141	0.016	
	Mod_cont_NL	BA decline + BA decline^2	5	-26.234	63.580	3.651	0.119	0.038	
**Average diameter at breast height of**	Mod_cont_NL	BA decline + BA decline^2	5	-182.940	377.303	0.000	0.949	0.007	No

**beech trees**	Mod_cont	BA decline	4	-187.300	383.531	6.228	0.042	0.003	
	Modnull1	Null model	3	-190.100	386.737	9.434	0.008	0.000	
**Average height of beech trees**	Mod_cont_NL	BA decline + BA decline^2	5	-150.090	311.599	0.000	0.907	0.046	No
	Mod_cont	BA decline	4	-153.720	316.376	4.777	0.083	0.044	
	Modnull1	Null model	3	-157.010	320.567	8.968	0.010	0.000	
**Volume of standing deadwood in a plot**	Mod_cont_NL	BA decline + BA decline^2	5	-606.230	1223.580	0.000	1.000	0.043	No
	Mod_cont	BA decline	4	-616.500	1241.730	18.148	0.000	0.042	
	Modnull1	Null model	3	-627.000	1260.420	36.843	0.000	0.000	
**Volume of lying deadwood in a plot**	Mod_cont_NL	BA decline + BA decline^2	5	-74.148	159.407	0.000	0.548	0.448	No
	Mod_cont	BA decline	4	-75.534	159.796	0.388	0.452	0.443	
	Modnull1	Null model	3	-93.483	193.394	33.987	0.000	0.000	
**C/N ratio of the soil**	Mod_cont_NL	BA decline + BA decline^2 + pH	5	-154.330	319.770	0.000	0.775	0.060	No
	Mod_cont	BA decline + pH	4	-156.800	322.325	2.555	0.216	0.056	
	Modnull1	Null model	3	-161.110	328.647	8.877	0.009	0.000	
**Potassium exchangeable cations concentration in the mineral layer**	Modnull1	Null model	3	76.590	-146.750	0.000	0.513	0.199	No
	Mod_cont	BA decline + pH	4	77.626	-146.530	0.225	0.458	0.317	

**soil**	Mod_cont_NL	BA decline + BA decline^2 + pH	5	76.036	-140.960	5.791	0.028	0.316	
**Magnesium exchangeable cations concentration in the mineral layer soil**	Mod_cont_NL	BA decline + BA decline^2 + pH	6	-105.070	223.724	0.000	0.546	0.035	No
	Mod_cont	BA decline + pH	5	-106.550	224.220	0.495	0.426	0.035	
	Modnull1	Null model	3	-111.600	229.631	5.907	0.028	0.000	
**Sodium exchangeable cations concentration in the mineral layer soil**	Mod_cont	BA decline + pH	5	110.275	-209.440	0.000	0.969	0.335	No
	Mod_cont_NL	BA decline + BA decline^2 + pH	6	107.980	-202.380	7.063	0.028	0.332	
	Modnull1	Null model	3	102.076	-197.720	11.715	0.003	0.000	
**Calcium exchangeable cations concentration in the mineral layer soil**	Mod_cont	BA decline + pH	5	17.362	-23.612	0.000	0.845	0.175	No
	Mod_cont_NL	BA decline + BA decline^2 + pH	6	16.642	-19.699	3.914	0.119	0.173	
	Modnull1	Null model	3	11.842	-17.256	6.356	0.035	0.000	
**Manganese exchangeable cations concentration in the mineral layer soil**	Modnull1	Null model	3	88.883	-171.340	0.000	0.983	0.065	No
	Mod_cont	BA decline + pH	4	85.913	-163.100	8.238	0.016	0.065	
	Mod_cont_NL	BA decline + BA decline^2 + pH	5	84.722	-158.330	13.006	0.001	0.085	

**Iron exchangeable cations concentration in the mineral layer soil**	Mod_cont_NL	BA decline + BA decline^2 + pH	5	-268.340	547.793	0.000	0.974	0.085	No
	Mod_cont	BA decline + pH	4	-273.180	555.087	7.294	0.025	0.072	
	Modnull1	Null model	3	-279.190	564.801	17.008	0.000	0.000	
**Aluminium exchangeable cations concentration in the mineral layer soil**	Modnull1	Null model	3	-38.524	83.476	0.000	0.511	0.000	No
	Mod_cont	BA decline + pH	4	-37.721	84.169	0.693	0.362	0.031	
	Mod_cont_NL	BA decline + BA decline^2 + pH	5	-37.576	86.262	2.786	0.127	0.031	
**Availability of soil phosphorus**	Modnull1	Null model	3	72.697	-138.970	0.000	0.982	0.000	No
	Mod_cont	BA decline + pH	4	69.793	-130.860	8.108	0.017	0.000	
	Mod_cont_NL	BA decline + BA decline^2 + pH	5	68.117	-125.120	13.844	0.001	0.000	
**Total soil nitrogen**	Modnull1	Null model	3	-61.364	129.156	0.000	0.931	0.000	No
	Mod_cont	BA decline + pH	5	-62.091	135.293	6.137	0.043	0.007	
	Mod_cont_NL	BA decline + BA decline^2 + pH	6	-61.363	136.312	7.156	0.026	0.009	
**Total soil carbon**	Mod_cont_NL	BA decline + BA decline^2 + pH	6	-228.010	469.603	0.000	0.943	0.076	No

	Mod_cont	BA decline + pH	5	-232.050	475.208	5.605	0.057	0.068	
	Modnull1	Null model	3	-240.080	486.589	16.986	0.000	0.000	
**Soil pH**	Modnull1	Null model	3	-16.753	39.934	0.000	0.853	0.000	No
	Mod_cont_NL	BA decline + BA decline^2	5	-16.862	44.835	4.901	0.074	0.037	
	Mod_cont	BA decline	4	-18.058	44.844	4.909	0.073	0.000	
**Electrical conductivity**	Modnull1	Null model	3	219.607	-432.790	0.000	0.996	0.105	No
	Mod_cont	BA decline	4	215.273	-421.820	10.966	0.004	0.136	
	Mod_cont_NL	BA decline + BA decline^2	5	213.517	-415.920	16.863	0.000	0.213	
**Net ammonification**	Modnull1	Null model	3	-88.247	182.964	0.000	0.484	0.047	No
	Mod_cont_NL	BA decline + BA decline^2	5	-86.432	184.088	1.125	0.276	0.052	
	Mod_cont	BA decline	4	-87.779	184.358	1.394	0.241	0.057	
**Net nitrification**	Mod_cont_NL	BA decline + BA decline^2	5	-90.104	191.433	0.000	0.531	0.104	No
	Mod_cont	BA decline	4	-91.485	191.770	0.337	0.449	0.103	
	Modnull1	Null model	3	-95.775	198.020	6.587	0.020	0.000	
**Net mineralisation**	Mod_cont_NL2	BA decline + BA decline^2 + pH	6	-118.420	250.589	0.000	0.532	0.069	No
	Mod_cont2	BA decline + pH	5	-120.620	252.466	1.877	0.208	0.064	
	Mod_cont_NL	BA decline + BA decline^2	5	-120.970	253.168	2.579	0.147	0.065	
	Mod_cont	BA decline	4	-123.250	255.303	4.715	0.050	0.056	

	Modnull1	Null model	3	-125.970	258.414	7.825	0.011	0.000	
**Soil respiration rate**	Mod_cont_NL	BA decline + BA decline^2	5	-80.996	173.100	0.000	0.684	0.155	Yes
	Mod_cont	BA decline	4	-84.043	176.800	3.710	0.216	0.103	
	Modnull1	Null model	3	-87.376	181.200	8.080	0.100	0.000	
**Soil temperature**	Mod_cont_NL	BA decline + BA decline^2	5	-99.623	210.356	0.000	0.739	0.136	No
	Mod_cont	BA decline	4	-101.860	212.443	2.087	0.260	0.122	
	Modnull1	Null model	3	-108.710	223.845	13.488	0.001	0.000	
**Total stand carbon (vegetation, deadwood and soil)**	Mod_cont	BA decline	4	266.419	-524.110	0.000	0.639	0.501	No
	Mod_cont_NL	BA decline + BA decline^2	5	267.038	-522.970	1.145	0.361	0.584	
	Modnull1	Null model	3	251.796	-497.160	26.946	0.000	0.000	
**Aboveground biomass**	Mod_cont	BA decline	4	-340.950	690.621	8.496	0.014	0.537	No
	Mod_cont_NL	BA decline + BA decline^2	5	-335.510	682.124	0.000	0.986	0.534	
	Modnull1	Null model	3	-372.150	750.723	68.599	0.000	0.000	
**Soil clay percentage**	Modnull1	Null model	3	-16.773	39.975	0.000	0.896	0.000	No
	Mod_cont	BA decline	4	-18.002	44.730	4.756	0.083	0.003	
	Mod_cont_NL	BA decline + BA decline^2	5	-18.164	47.439	7.465	0.021	0.004	
**Soil silt percentage**	Modnull1	Null model	3	2.618	1.193	0.000	0.718	0.000	No
	Mod_cont	BA decline	4	2.658	3.411	2.218	0.237	0.043	

	Mod_cont_NL	BA decline + BA decline^2	5	2.195	6.721	5.528	0.045	0.043	
**Soil sand percentage**	Modnull1	Null model	3	-20.488	47.404	0.000	0.823	0.000	No
	Mod_cont	BA decline	4	-21.116	50.958	3.554	0.139	0.014	
	Mod_cont_NL	BA decline + BA decline^2	5	-21.213	53.536	6.133	0.038	0.014	
**Bracken cover**	Mod_cont_NL	BA decline + BA decline^2	5	-137.020	285.155	0.000	0.711	0.245	No
	Mod_cont	BA decline	4	-139.110	286.952	1.797	0.289	0.245	
	Modnull1	Null model	3	-150.300	307.035	21.880	0.000	0.000	
**Bare ground and moss cover**	Mod_cont_NL	BA decline + BA decline^2	5	-101.160	213.425	0.000	0.769	0.199	No
	Mod_cont	BA decline	4	-103.560	215.847	2.422	0.229	0.175	
	Modnull1	Null model	3	-109.540	225.517	12.092	0.002	0.000	
**Litter cover**	Mod_cont_NL	BA decline + BA decline^2	5	-119.170	249.446	0.000	0.718	0.646	No
	Mod_cont	BA decline	4	-121.300	251.319	1.873	0.282	0.645	
	Modnull1	Null model	3	-159.070	324.574	75.129	0.000	0.000	
**Grass cover**	Mod_cont	BA decline	4	9.434	-10.140	0.000	0.819	0.161	No
	Mod_cont_NL	BA decline + BA decline^2	5	9.080	-7.049	3.091	0.175	0.164	
	Modnull1	Null model	3	3.389	-0.350	9.790	0.006	0.000	
**Palatable tree browseline**	Mod_cont_NL	BA decline + BA decline^2	5	-94.720	200.979	0.000	0.556	0.028	No
	Mod_cont	BA decline	4	-96.760	202.519	1.541	0.257	0.028	

	Modnull1	Null model	3	-98.285	203.155	2.176	0.187	0.000	
**Unpalatable tree browseline**	Mod_cont_NL	BA decline + BA decline^2	5	-112.050	235.380	0.000	0.602	0.035	No
	Mod_cont	BA decline	4	-114.080	237.002	1.622	0.268	0.031	
	Modnull1	Null model	3	-115.980	238.449	3.069	0.130	0.000	
**Holly cover**	Modnull1	Null model	3	-66.398	139.445	0.000	0.471	0.000	No
	Mod_cont_NL	BA decline + BA decline^2	5	-64.272	140.258	0.813	0.313	0.005	
	Mod_cont	BA decline	4	-65.945	141.002	1.557	0.216	0.002	
**Rubus cover**	Mod_cont_NL	BA decline + BA decline^2	5	-71.326	154.366	0.000	0.622	0.184	No
	Mod_cont	BA decline	4	-73.140	155.391	1.025	0.373	0.188	
	Modnull1	Null model	3	-78.591	163.832	9.466	0.005	0.000	
**Holly shrubs browsed**	Modnull1	Null model	3	-58.867	124.163	0.000	0.407	0.000	No
	Mod_cont	BA decline	4	-57.975	124.677	0.514	0.315	0.047	
	Mod_cont_NL	BA decline + BA decline^2	5	-56.907	124.926	0.763	0.278	0.059	
**Rubus shrubs browsed**	Mod_cont_NL	BA decline + BA decline^2	5	-73.077	157.868	0.000	0.831	0.129	No
	Mod_cont	BA decline	4	-76.250	161.611	3.744	0.128	0.076	
	Modnull1	Null model	3	-78.612	163.873	6.005	0.041	0.000	
**Average crown condition**	Mod_cont	BA decline	4	9.554	-10.177	0.000	0.639	0.156	No
	Mod_cont_NL	BA decline + BA decline^2	5	9.691	-7.954	2.224	0.210	0.155	
	Modnull1	Null model	3	6.921	-7.296	2.881	0.151	0.000	
**Understorey**	Modnull1	Null model	3	-19.867	46.350	0.000	0.829	0.000	No

**condition**	Mod_cont	BA decline	4	-20.713	50.478	4.128	0.105	0.004	
	Mod_cont_NL	BA decline + BA decline^2	5	-19.898	51.418	5.068	0.066	0.028	
**Canopy openness**	Mod_cont_NL	BA decline + BA decline^2	5	-43.877	98.866	0.000	0.988	0.886	Yes
	Mod_cont	BA decline	4	-49.514	107.756	8.890	0.012	0.872	
	Modnull1	Null model	3	-112.800	232.025	133.159	0.000	0.000	
**Understorey openness**	Mod_cont_NL	BA decline + BA decline^2	5	-115.730	242.573	0.000	0.602	0.292	No
	Mod_cont	BA decline	4	-117.340	243.401	0.828	0.398	0.295	
	Modnull1	Null model	3	-130.790	268.004	25.431	0.000	0.000	
**Tree seedling richness**	Mod_cont	BA decline	3	-102.420	211.273	0.000	0.732	0.195	No
	Mod_cont_NL	BA decline + BA decline^2	4	-102.290	213.301	2.028	0.265	0.209	
	Modnull1	Null model	2	-109.100	222.414	11.141	0.003	0.000	
**Tree sapling richness**	Modnull1	Null model	2	-62.582	129.375	0.000	0.693	0.000	No
	Mod_cont	BA decline	3	-62.561	131.551	2.176	0.233	0.001	
	Mod_cont_NL	BA decline + BA decline^2	4	-62.561	133.850	4.475	0.074	0.001	
**Spider species richness**	Mod_cont	BA decline	3	-55.813	118.769	0.000	0.496	0.138	No
	Modnull1	Null model	2	-57.636	119.817	1.048	0.294	0.000	
	Mod_cont_NL	BA decline + BA decline^2	4	-55.245	120.490	1.721	0.210	0.189	
**Rove beetles species richness**	Modnull1	Null model	2	-50.365	105.276	0.000	0.595	0.000	No
	Mod_cont_NL	BA decline	4	-48.635	107.270	1.994	0.220	0.134	

		+ BA decline^2						
	Mod_cont	BA decline	3	-50.232	107.607	2.331	0.185	0.012	
**Carabid beetles species richness**	Modnull1	Null model	2	-51.530	107.606	0.000	0.614	0.000	No
	Mod_cont	BA decline	3	-51.005	109.153	1.547	0.283	0.046	
	Mod_cont_NL	BA decline + BA decline^2	4	-50.590	111.179	3.573	0.103	0.086	
**Ant species richness**	Mod_cont	BA decline	3	-37.656	82.455	0.000	0.775	0.484	No
	Mod_cont_NL	BA decline	4	-37.467	84.933	2.479	0.224	0.529	
	Modnull1	Null model	2	-45.428	95.401	12.946	0.001	0.000	
**Weevil species richness**	Modnull1	Null model	2	-28.533	61.611	0.000	0.724	0.000	No
	Mod_cont	BA decline	3	-28.485	64.113	2.502	0.207	0.006	
	Mod_cont_NL	BA decline + BA decline^2	4	-28.165	66.330	4.719	0.068	0.048	
**Woodlice species richness**	Modnull1	Null model	2	-37.242	79.029	0.000	0.732	0.000	No
	Mod_cont	BA decline	3	-37.226	81.595	2.566	0.203	0.002	
	Mod_cont_NL	BA decline + BA decline^2	4	-36.943	83.887	4.857	0.065	0.029	
**Ground-dwelling arthropod species richness**	Mod_cont	BA decline	3	-69.500	146.150	0.000	0.740	0.264	No
	Mod_cont_NL	BA decline + BA decline^2	4	-69.280	148.560	2.410	0.220	0.283	

	Modnull1	Null model	2	-73.720	151.980	5.840	0.040	0.000	
**Moisture content of the mineral layer**	Mod_cont_NL	BA decline + BA decline^2	5	-208.680	428.469	0.000	0.909	0.026	No
	Mod_cont	BA decline	4	-212.410	433.539	5.070	0.072	0.013	
	Modnull1	Null model	3	-214.890	436.202	7.733	0.019	0.000	
**Moisture content of the organic layer**	Mod_cont_NL	BA decline + BA decline^2	5	-300.810	612.734	0.000	0.971	0.005	No
	Mod_cont	BA decline	4	-305.580	619.878	7.143	0.027	0.005	
	Modnull1	Null model	3	-309.380	625.194	12.460	0.002	0.000	
**Cervus dung proportional**	Mod_cont_NL	BA decline + BA decline^2	4	-2758.300	5525.300	0.000	1.000	0.029	No
	Mod_cont	BA decline	3	-2780.300	5567.070	41.766	0.000	0.001	
	Modnull1	Null model	2	-2871.200	5746.540	221.241	0.000	0.000	
**Equus dung proportional**	Mod_cont	BA decline	3	-627.110	1260.650	0.000	0.759	0.173	No
	Mod_cont_NL	BA decline + BA decline^2	4	-627.110	1262.950	2.298	0.241	0.175	
	Modnull1	Null model	2	-729.680	1463.570	202.920	0.000	0.000	
**Proportional dung total**	Mod_cont_NL	BA decline + BA decline^2	4	-2636.600	5281.920	0.000	1.000	0.016	No
	Mod_cont	BA decline	3	-2647.100	5300.560	18.636	0.000	0.004	
	Modnull1	Null model	2	-2674.300	5352.800	70.880	0.000	0.000	
**Very large beech**	Mod_cont	BA decline	3	-61.549	129.643	0.000	0.586	0.104	No

**trees (74.97 cm < dbh < 103 cm)**	Modnull1	Null model	2	-63.607	131.480	1.836	0.234	0.000	
	Mod_cont_NL	BA decline + BA decline^2	4	-61.535	132.000	2.356	0.180	0.101	
**Large beech trees (68.32 cm < dbh < 74.97 cm)**	Mod_cont	BA decline	3	-59.977	126.499	0.000	0.744	0.294	No
	Mod_cont_NL	BA decline + BA decline^2	4	-59.857	128.644	2.145	0.255	0.322	
	Modnull1	Null model	2	-67.724	139.714	13.216	0.001	0.000	
**Holly tree abundance**	Mod_cont	BA decline	3	-118.510	243.555	0.000	0.454	0.015	No
	Mod_cont_NL	BA decline + BA decline^2	4	-117.800	244.532	0.978	0.279	0.019	
	Modnull1	Null model	2	-120.170	244.615	1.060	0.267	0.000	
**Beech trees abundance**	Mod_cont_NL	BA decline + BA decline^2	4	-101.000	210.719	0.000	1.000	0.778	Yes
	Mod_cont	BA decline	3	-111.490	229.400	18.682	0.000	0.639	
	Modnull1	Null model	2	-171.050	346.306	135.587	0.000	0.000	
**Holly saplings abundance**	Mod_cont_NL	BA decline + BA decline^2	4	-354.540	717.797	0.000	0.988	0.005	No
	Mod_cont	BA decline	3	-360.280	726.991	9.195	0.010	0.000	
	Modnull1	Null model	2	-363.170	730.549	12.752	0.002	0.000	
**Beech saplings abundance**	Mod_cont_NL	BA decline + BA decline^2	4	-35.653	80.033	0.000	0.997	0.075	No
	Mod_cont	BA decline	3	-42.921	92.270	12.236	0.002	0.008	
	Modnull1	Null model	2	-44.862	93.935	13.902	0.001	0.000	

**Overall saplings abundance**	Mod_cont_NL	BA decline + BA decline^2	4	-369.570	747.876	0.000	0.950	0.006	No
	Mod_cont	BA decline	3	-373.970	754.369	6.493	0.037	0.000	
	Modnull1	Null model	2	-376.150	756.504	8.628	0.013	0.000	
**Ground flora species richness**	Mod_cont_NL	BA decline + BA decline^2 + log(Dung)	5	-183.090	377.285	0.000	0.898	0.596	Yes
	Mod_cont	BA decline + log(Dung)	4	-186.960	382.653	5.368	0.061	0.548	
	Mod_cont_NL2	BA decline + BA decline^2	4	-187.400	383.531	6.246	0.040	0.549	
	Mod_cont2	BA decline	3	-192.550	391.533	14.249	0.001	0.486	
	Modnull1	Null model	3	-257.450	521.336	144.052	0.000	0.028	
**Woody ground flora species richness**	Mod_cont2	BA decline	3	-112.510	231.446	0.000	0.494	0.052	No
	Mod_cont	BA decline + log(Dung)	4	-112.400	233.532	2.087	0.174	0.055	
	Mod_cont_NL2	BA decline + BA decline^2	4	-112.500	233.731	2.285	0.158	0.053	
	Modnull1	Null model	3	-113.920	234.265	2.819	0.121	0.001	
	Mod_cont_NL	BA decline + BA decline^2 + log(Dung)	5	-112.400	235.912	4.467	0.053	0.056	
**Non-woody ground flora species richness**	Mod_cont_NL	BA decline + BA decline^2 + log(Dung)	5	-172.810	356.738	0.000	0.956	0.655	Yes

	Mod_cont_NL2	BA decline + BA decline^2	4	-177.130	362.980	6.242	0.042	0.610	
	Mod_cont	BA decline + log(Dung)	4	-180.150	369.033	12.295	0.002	0.582	
	Mod_cont2	BA decline	3	-186.090	378.598	21.860	0.000	0.517	
	Modnull1	Null model	3	-262.040	530.507	173.769	0.000	0.032	
**Lichen species richness**	Mod_cont_NL	BA decline + BA decline^2 + Holly abundance	5	-221.100	453.317	0.000	1.000	0.437	Yes
	Mod_cont	BA decline + Holly abundance	4	-231.850	472.417	19.100	0.000	0.331	
	Modnull1	Null model	3	-250.110	506.652	53.335	0.000	0.140	
**Lichen species richness on holly**	Modnull1	Null model	2	-224.964	454.138	0.000	0.498	0.000	No
	Mod_cont	BA decline	3	-224.168	454.764	0.626	0.364	0.001	
	Mod_cont_NL	BA decline + BA decline^2	4	-223.993	456.712	2.574	0.138	0.004	
**Lichen species richness on beech**	Mod_cont_NL	BA decline + BA decline^2	4	-208.980	426.688	0.000	1.000	0.599	Yes
	Mod_cont	BA decline	3	-238.790	484.014	57.326	0.000	0.392	
	Modnull1	Null model	2	-289.570	583.340	156.652	0.000	0.000	
**Organic layer loss on ignition**	Modnull1	Null model	3	-47.462	101.352	0.000	0.735	0.000	No
	Mod_cont	BA decline	4	-47.661	104.049	2.697	0.191	0.008	
	Mod_cont_NL	BA decline + BA decline^2	5	-47.408	105.927	4.575	0.075	0.008	

**Mineral layer loss on ignition**	Modnull1	Null model	3	-63.385	133.199	0.000	0.520	0.000	No
	Mod_cont	BA decline	4	-62.741	134.209	1.010	0.314	0.020	
	Mod_cont_NL	BA decline + BA decline^2	5	-62.180	135.470	2.271	0.167	0.020	
**Organic layer nitrate concentration**	Modnull1	Null model	3	-63.091	132.611	0.000	0.399	0.000	No
	Mod_cont_NL	BA decline + BA decline^2	5	-60.917	132.946	0.335	0.338	0.054	
	Mod_cont	BA decline	4	-62.359	133.446	0.835	0.263	0.034	
**Mineral layer nitrate concentration**	Modnull1	Null model	3	-63.091	132.611	0.000	0.399	0.000	No
	Mod_cont_NL	BA decline + BA decline^2	5	-60.917	132.946	0.335	0.338	0.054	
	Mod_cont	BA decline	4	-62.359	133.446	0.835	0.263	0.034	
**Organic layer ammonium concentration**	Mod_cont_NL	BA decline + BA decline^2	5	-235.070	481.246	0.000	0.959	0.052	No
	Mod_cont	BA decline	4	-239.470	487.665	6.419	0.039	0.036	
	Modnull1	Null model	3	-243.470	493.374	12.128	0.002	0.000	
**Mineral layer ammonium concentration**	Modnull1	Null model	3	-43.781	93.990	0.000	0.776	0.000	No
	Mod_cont	BA decline	4	-44.375	97.477	3.487	0.136	0.003	
	Mod_cont_NL	BA decline + BA decline^2	5	-43.620	98.351	4.361	0.088	0.006	
**Potentially mineralisable nitrogen of the organic layer**	Modnull1	Null model	3	-122.240	250.909	0.000	0.461	0.000	No
	Mod_cont_NL	BA decline + BA decline^2	5	-120.250	251.611	0.702	0.325	0.001	
	Mod_cont	BA decline	4	-121.860	252.438	1.529	0.215	0.001	

**Potentially mineralisable nitrogen of the mineral layer**	Mod_cont_NL	BA decline<br>+ BA decline^2<br>+ soil moisture	6	-186.840	387.270	0.000	0.974	0.129	No
	Mod_cont	BA decline<br>+ soil moisture	5	-191.740	394.586	7.317	0.025	0.091	
	Modnull1	Null model	4	-196.920	402.558	15.289	0.000	0.014	
**Understorey biomass**	Mod_cont_NL	BA decline<br>+ BA decline^2	6	-137.210	288.010	0.000	0.905	0.380	Yes
	Mod_cont	BA decline	5	-141.355	293.820	5.810	0.050	0.342	
	Modnull1	Null model	4	-142.626	293.980	5.970	0.046	0.335	

Table S4: Updated version of Table S3 with only linear and quadratic term of BA included as fixed effects.

Response variable	Name	Model structure	df	Log likelihood	AICc	ΔAICc	AICc weight	Marginal r^2	Threshold?
**Abundance of holly seedlings**	Mod_cont_NL	BA decline + BA decline^2	4	-1364.378	2737.483	0.000	1.000	0.116	No
	Mod_cont	BA decline	3	-1849.403	3705.234	967.751	0.000	0.033	
	Modnull1	Null model	2	-1895.355	3794.921	1057.438	0.000	0.000	
**Abundance of beech seedlings**	Mod_cont_NL	BA decline + BA decline^2	4	-279.394	567.515	0.000	1.000	0.217	Yes
	Mod_cont	BA decline	3	-302.158	610.744	43.229	0.000	0.170	
	Modnull1	Null model	2	-331.657	667.524	100.009	0.000	0.000	
**Abundance of oak seedlings**	Mod_cont_NL	BA decline+ BA decline^2	4	-50.284	109.295	0.000	0.999	0.444	Yes
	Mod_cont	BA decline	3	-58.639	123.706	14.412	0.001	0.147	
	Modnull1	Null model	2	-65.866	135.942	26.648	0.000	0.000	
**Abundance of tree seedlings**	Mod_cont_NL	BA decline+ BA decline^2	4	-1403.461	2815.650	0.000	1.000	0.134	No
	Mod_cont	BA decline	3	-1907.548	3821.524	1005.874	0.000	0.046	
	Modnull1	Null model	2	-1970.624	3945.459	1129.809	0.000	0.000	
**Abundance of palatable**	Mod_cont_NL	BA decline + BA decline^2	4	-267.337	543.401	0.000	1.000	0.293	Yes

**seedlings**	Mod_cont	BA decline	3	-296.268	598.964	55.564	0.000	0.224	
	Modnull1	Null model	2	-332.499	669.209	125.808	0.000	0.000	
	Mod_cont	BA decline	4	-75.534	159.796	0.388	0.452	0.443	
	Modnull1	Null model	3	-93.483	193.394	33.987	0.000	0.000	
**C/N ratio of the soil**	Mod_cont_NL	BA decline + BA decline^2	5	-154.329	319.770	0.000	0.775	0.060	No
	Mod_cont	BA decline	4	-156.799	322.325	2.555	0.216	0.056	
	Modnull1	Null model	3	-161.109	328.647	8.877	0.009	0.000	
**Potassium exchangeable cations concentration in the mineral layer soil**	Modnull1	Null model	3	76.590	-146.751	0.000	0.513	0.000	No
	Mod_cont	BA decline	4	77.626	-146.525	0.225	0.458	0.099	
	Mod_cont_NL	BA decline + BA decline	5	76.035	-140.960	5.791	0.028	0.102	
**Magnesium exchangeable cations concentration in the mineral layer soil**	Mod_cont_NL	BA decline + BA decline^2	5	-109.120	229.352	0.000	0.380	0.018	No
	Mod_cont	BA decline	3	-111.601	229.631	0.279	0.330	0.000	
	Modnull1	Null model	4	-110.582	229.891	0.539	0.290	0.018	
**Sodium exchangeable cations**	Mod_cont	BA decline	4	112.188	-215.649	0.000	0.971	0.339	No

**concentration in the mineral layer soil**	Mod_cont_NL	BA decline + BA decline^2	5	109.859	-208.606	7.043	0.029	0.336	
	Modnull1	Null model	3	102.076	-197.722	17.926	0.000	0.000	
**Calcium exchangeable cations concentration in the mineral layer soil**	Mod_cont_NL	BA decline + BA decline^2	5	15.602	-20.092	0.000	0.805	0.141	No
	Modnull1	Null model	3	11.842	-17.256	2.836	0.195	0.000	
	Mod_cont	BA decline	4	-123.252	255.303	275.395	0.000	0.056	
**Manganese exchangeable cations concentration in the mineral layer soil**	Modnull1	Null model	3	88.883	-171.338	0.000	0.983	0.000	No
	Mod_cont	BA decline	4	85.913	-163.100	8.238	0.016	0.003	
	Mod_cont_NL	BA decline + BA decline^2	5	84.722	-158.333	13.005	0.001	0.024	
**Iron exchangeable cations concentration in the mineral layer soil**	Mod_cont_NL	BA decline + BA decline	5	-268.341	547.793	0.000	0.974	0.085	No
	Mod_cont	BA decline	4	-273.180	555.087	7.294	0.025	0.072	
	Modnull1	Null model	3	-279.186	564.801	17.008	0.000	0.000	
**Aluminium exchangeable cations concentration in the mineral layer soil**	Modnull1	Null model	3	-38.524	83.476	0.000	0.511	0.000	No
	Mod_cont	BA decline	4	-37.721	84.169	0.693	0.362	0.031	
	Mod_cont_NL	BA decline + BA decline^2	5	-37.576	86.262	2.786	0.127	0.031	
**Availability of soil phosphorus**	Modnull1	Null model	3	72.697	-138.966	0.000	0.982	0.000	No

	Mod_cont	BA decline	4	69.793	-130.859	8.108	0.017	0.000	
	Mod_cont_NL	BA decline + BA decline^2	5	68.117	-125.122	13.844	0.001	0.000	
**Total soil nitrogen**	Modnull1	Null model	3	-61.364	129.156	0.000	0.773	0.000	No
	Mod_cont	BA decline	4	-61.891	132.510	3.354	0.144	0.002	
	Mod_cont_NL	BA decline + BA decline^2	5	-61.260	133.631	4.475	0.083	0.003	
**Total soil carbon**	Mod_cont_NL	BA decline + BA decline^2	5	-230.653	472.418	0.000	0.943	0.077	No
	Mod_cont	BA decline	4	-234.674	478.076	5.658	0.056	0.069	
	Modnull1	Null model	3	-240.080	486.589	14.171	0.001	0.000	
**Net mineralisation**	Mod_cont_NL	BA decline + BA decline^2	5	-120.972	253.168	0.000	0.706	0.065	No
	Mod_cont	BA decline	4	-123.252	255.303	2.135	0.243	0.056	
	Modnull1	Null model	5	-125.972	258.414	5.246	0.051	0.000	
**Ground flora species richness**	Mod_cont_NL	BA decline + BA decline^2	4	-187.402	383.531	0.000	0.982	0.549	Yes
	Mod_cont	BA decline	3	-192.552	391.533	8.002	0.018	0.486	
	Modnull1	Null model	3	-257.751	519.712	136.181	0.000	0.000	
**Woody ground flora species richness**	Mod_cont	BA decline	3	-112.508	231.446	0.000	0.491	0.052	No
	Modnull1	Null model	2	-113.948	232.107	0.662	0.353	0.000	

	Mod_cont_NL	BA decline + BA decline^2	4	-112.502	233.731	2.285	0.157	0.053	
**Non-woody ground flora species richness**	Mod_cont_NL	BA decline + BA decline^2	4	-177.126	362.979	0.000	1.000	0.610	Yes
	Mod_cont	BA decline + BA decline^2	3	-186.085	378.598	15.618	0.000	0.517	
	Modnull1	BA decline	2	-262.197	528.604	165.624	0.000	0.000	
**Lichen species richness**	Mod_cont_NL	BA decline + BA decline^2	4	-243.059	494.845	0.000	0.998	0.240	Yes
	Mod_cont	BA decline	3	-250.311	507.050	12.205	0.002	0.169	
	Modnull1	Null model	2	-265.919	536.048	41.203	0.000	0.000	
**Potentially mineralisable nitrogen of the mineral layer**	Mod_cont_NL	BA decline + BA decline^2	5	-185.964	383.038	0.000	0.982	0.114	No
	Mod_cont	BA decline	4	-191.192	391.112	8.074	0.017	0.068	
	Modnull1	Null model	3	-195.963	398.355	15.317	0.000	0.000	

Table S5: Statistics of the soil properties. Mean, standard deviation (SD), standard error (SE), and confidence interval (CI) of several soil properties across the stages of dieback.

	**Percent basal area decline**	N	Mean	SD	SE	CI
Clay (%)	0%	12	20.42	3.68	1.06	2.34
	25%	12	20.00	4.75	1.37	3.02
	50%	12	21.08	7.29	2.11	4.63
	75%	12	19.08	6.24	1.80	3.97
	100%	12	20.58	7.90	2.28	5.02
Sand (%)	0%	12	48.83	6.79	1.96	4.32
	25%	12	49.50	6.47	1.87	4.11
	50%	12	49.50	10.12	2.92	6.43
	75%	12	52.50	10.98	3.17	6.97
	100%	12	51.08	10.40	3.00	6.61
Silt (%)	0%	12	30.75	4.81	1.39	3.05
	25%	12	30.50	4.52	1.31	2.87
	50%	12	29.42	4.87	1.41	3.09
	75%	12	28.42	5.68	1.64	3.61
	100%	12	28.33	4.21	1.21	2.67
pH	0%	12	4.19	0.28	0.08	0.18
	25%	12	4.40	0.38	0.11	0.24
	50%	12	4.37	0.28	0.08	0.18
	75%	12	4.27	0.27	0.08	0.17
	100%	12	4.27	0.35	0.10	0.23
Moisture content (Organic layer)	0%	12	157.07	41.05	11.85	26.08
	25%	12	163.33	50.04	14.45	31.80
	50%	12	149.21	53.35	15.40	33.89

	75%	12	153.40	53.37	15.41	33.91
	100%	12	149.42	67.39	19.45	42.82
Moisture content (Mineral layer)	0%	12	27.94	4.85	1.40	3.08
	25%	12	34.58	16.45	4.75	10.45
	50%	12	29.00	4.76	1.37	3.02
	75%	12	27.68	6.67	1.93	4.24
	100%	12	27.81	5.57	1.61	3.54

Supplementary Methods: SM3. Graphs to support the space-for-time assumption

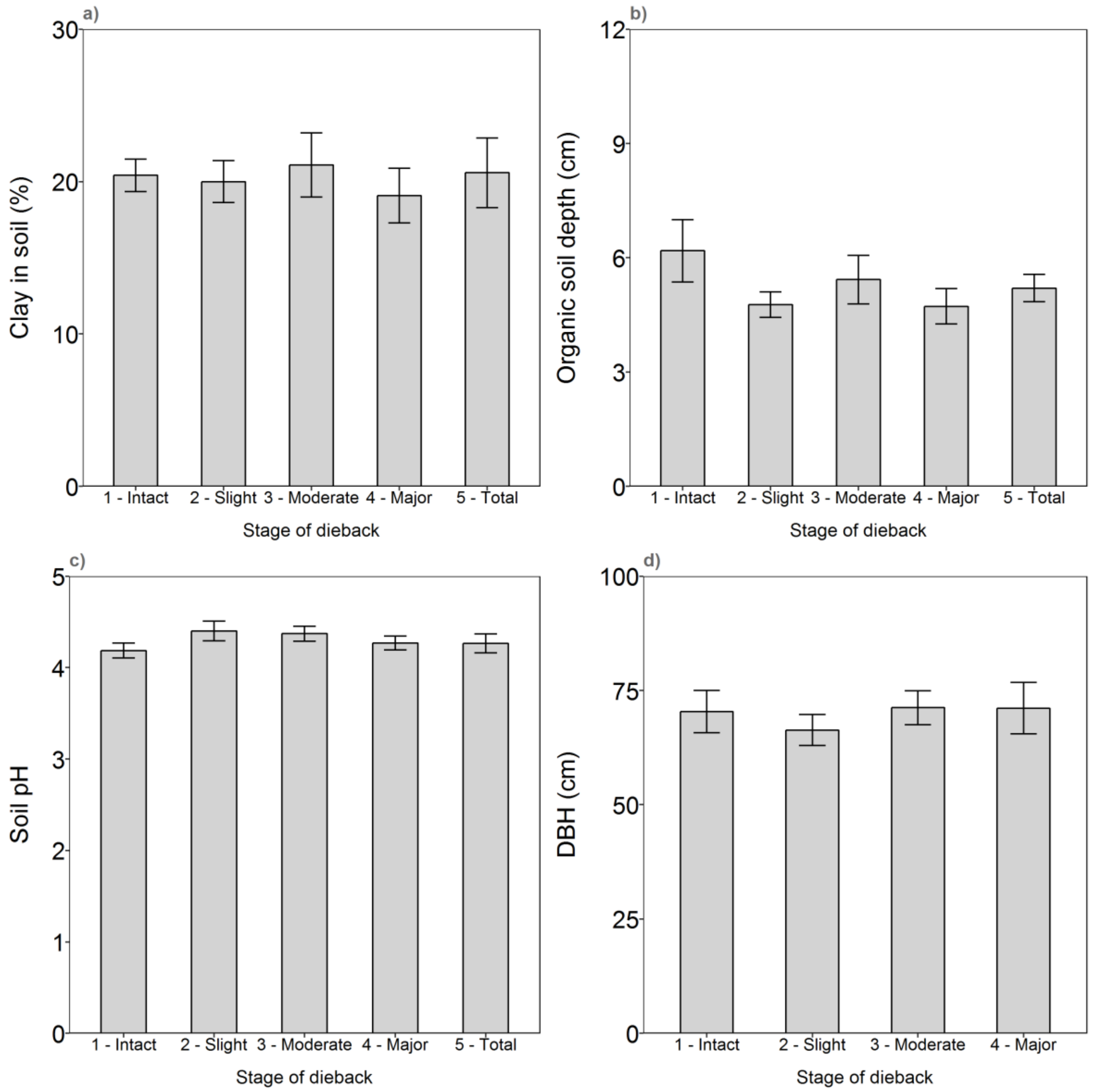

Fig S3: Mean values (n = 12) of a) clay soil content; b) depth of the organic soil layer; c) pH of the soil across the gradient of dieback; and d) diameter at breast height (DBH) of the living beech trees across the gradient of dieback. The black bars indicate the standard error of the mean.

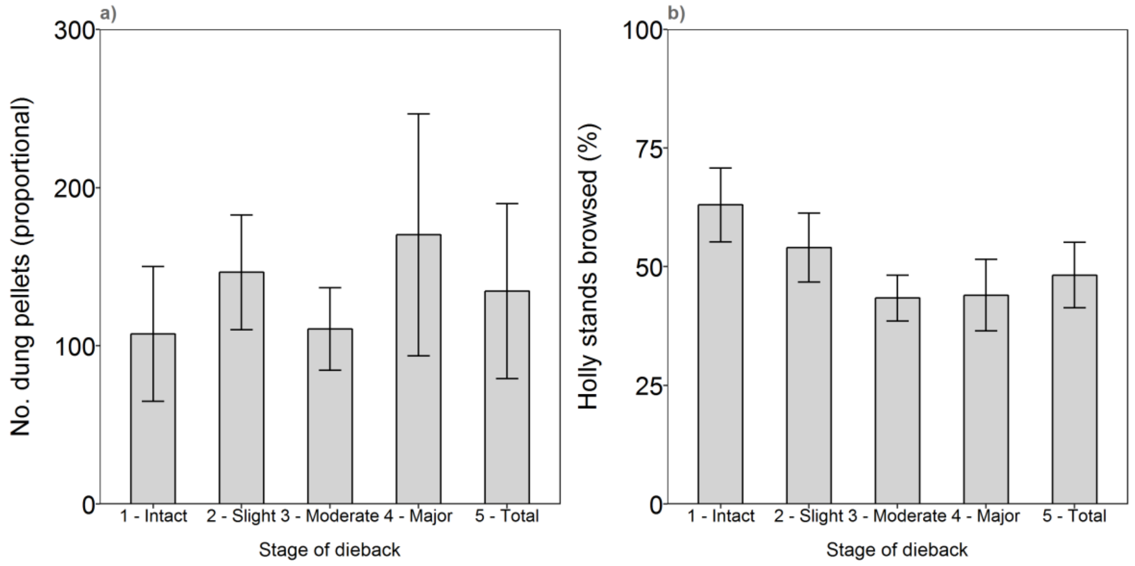

Fig S4: Mean values of a) the total herbivore dung count, and b) percentage of holly shoots browsed by herbivores across the gradient of dieback. The black bars indicate the standard error of the mean.

Supplementary references

1. Husch, B., Beers, T. W. & Kershaw, J. A. *Forest mensuration* (Wiley, 2003).
2. van Laar, A. & Akça, A. *Forest mensuration* (Springer, 2007).
3. Cantarello, E. & Newton, A. C. Identifying cost-effective indicators to assess the conservation status of forested habitats in Natura 2000 sites. *For. Ecol. Manage.* **256**, 815-826, doi:10.1016/j.foreco.2008.05.031 (2008).
4. Strickler, G. S. Use of the densiometer to estimate density of forest canopy on permanent sample plots (USDA Forest Service, 1959).
5. Jenkins, T. A. R. *et al.* FC woodland carbon code: Carbon assessment protocol (Forestry Commission, 2011).
6. McKay, H., Hudson, J. B. & Hudson, R. J. Woodfuel resource in Britain: Appendices. Fes b/w3/00787/rep/2. Dti/pub urn 03/1436 (Forestry Contracting Association, 2003).
7. Matthews, G. A. R. The carbon content of trees. Forestry commission technical paper 4 (Forestry Commission, 1993).
8. Stewart, K. E. J., Bourn, N. A. D. & Thomas, J. A. An evaluation of three quick methods commonly used to assess sward height in ecology. *J. Appl. Ecol.* **38**, 1148-1154, doi:10.1046/j.1365-2664.2001.00658.x (2001).
9. Bergström, R. & Guillet, C. Summer browsing by large herbivores in short-rotation willow plantations. *Biomass Bioenergy* **23**, 27-32, doi:10.1016/S0961-9534(02)00027-2 (2002).
10. Gibson, D. J. *Methods in comparative plant population ecology* (Oxford University Press, 2002).
11. Bazely, D. R., Myers, J. H. & da Silva, K. B. The response of numbers of bramble prickles to herbivory and depressed resource availability. *Oikos* **61**, 327-336, doi:10.2307/3545240 (1991).
12. Campbell, D., Swanson, G. M. & Sales, J. Methodological insights: Comparing the precision and cost-effectiveness of faecal pellet group count methods. *J. Appl. Ecol.* **41**, 1185-1196, doi:10.1111/j.0021-8901.2004.00964.x (2004).

13. Marques, F. F. C. *et al.* Estimating deer abundance from line transect surveys of dung: Sika deer in southern Scotland. *J. Appl. Ecol.***38**, 349-363, doi:10.1046/j.1365-2664.2001.00584.x (2001).
14. Jenkins, K. J. & Manly, B. A double-observer method for reducing bias in faecal pellet surveys of forest ungulates. *J. Appl. Ecol.***45**, 1339-1348, doi:10.1111/j.1365-2664.2008.01512.x (2008).
15. DeLuca, T., Zewdie, S., Zackrisson, O., Healey, J. & Jones, D. Bracken fern (*Pteridium aquilinum* L. Kuhn) promotes an open nitrogen cycle in heathland soils. *Plant Soil* **367**, 521-534, doi:10.1007/s11104-012-1484-0 (2013).
16. Mulvaney, R. S. Nitrogen - inorganic forms in *Methods of soil analysis. Part 3 - chemical methods* (ed. Sparks, D. L.) 1123–1184 (Soil Science Society of America, 1996).
17. Miranda, K. M., Espey, M. G. & Wink, D. A. A rapid, simple spectrophotometric method for simultaneous detection of nitrate and nitrite. *Nitric Oxide* **5**, 62-71, doi:10.1006/niox.2000.0319 (2001).
18. Keeney, D. R. in *Methods of soil analysis: Chemical and microbiological properties* (eds. Page, A. L., Miller, R. H. & Keeney, D. R.) (Soil Society of America, 1982).
19. PP Systems. EGM-4 Environmental Gas Monitor for CO_2: Operator's manual version 4.18 (PP Systems, 2010).
20. Rowell, D. L. *Soil science: Methods & application* (John Wiley & Sons, Ltd, 1994).
21. Dytham, C. *Choosing and using statistics: A biologist's guide* (John Wiley & Sons, 2011).
22. Warton, D. I. & Hui, F. K. C. The arcsine is asinine: The analysis of proportions in ecology. *Ecology* **92**, 3-10, doi:10.1890/10-0340.1 (2010).
23. Burnham, K. P. & Anderson, D. R. *Model selection and multimodel inference* (Springer-Verlag, 2002).
24. Barton, K. Mumin: Multi-model inference: R package version 1.10.0. (2014).
25. Nakagawa, S. & Schielzeth, H. A general and simple method for obtaining R^2 from generalized linear mixed-effects models. *Methods Ecol. Evol.* **4**, 133-142 (2013).
26. Yu, D. W. *et al.* Biodiversity soup: Metabarcoding of arthropods for rapid biodiversity assessment and biomonitoring. *Methods Ecol. Evol.* **3**, 613-623, doi:10.1111/j.2041-210X.2012.00198.x (2012).
27. NBN Gateway. National Biodiversity Networks Gateway https://data.nbn.org.uk/ (2015).
28. de Jong, Y. et al. Fauna Europaea – all European animal species on the web. Biodiversity Data Journal. **2**, e4034, doi:10.3897/BDJ.2.e4034 (2014).
29. AntWeb. AntWeb https://www.antweb.org (2015)
30. British Arachnological Society. Spider and harvestman recording scheme http://srs.britishspiders.org.uk (2015).
31. Nentwig W, Blick T, Gloor D, Hänggi A, Kropf C. Spiders of Europe www.araneae.unibe.ch (2015).
